# Mapping Nanoscale‐To‐Single‐Cell Phosphoproteomic Landscape by Chip‐DIA

**DOI:** 10.1002/advs.202402421

**Published:** 2024-10-14

**Authors:** Gul Muneer, Sofani Tafesse Gebreyesus, Ciao‐Syuan Chen, Tzu‐Tsung Lee, Fengchao Yu, Chih‐An Lin, Min‐Shu Hsieh, Alexey I. Nesvizhskii, Chao‐Chi Ho, Sung‐Liang Yu, Hsiung‐Lin Tu, Yu‐Ju Chen

**Affiliations:** ^1^ Institute of Chemistry Academia Sinica Taipei 115201 Taiwan; ^2^ Institute of Biochemical Sciences National Taiwan University Taipei 106319 Taiwan; ^3^ Chemical Biology and Molecular Biophysics Program Taiwan International Graduate Program Academia Sinica Taipei 11529 Taiwan; ^4^ Department of Pathology University of Michigan Ann Arbor MI 48109 USA; ^5^ Department of Internal Medicine National Taiwan University Hospital Taipei 10051 Taiwan; ^6^ Department of Pathology National Taiwan University Cancer Center Taipei 10617 Taiwan; ^7^ Department of Pathology National Taiwan University Hospital Taipei 100225 Taiwan; ^8^ Graduate Institute of Pathology National Taiwan University College of Medicine Taipei 10051 Taiwan; ^9^ Department of Computational Medicine and Bioinformatics University of Michigan Ann Arbor MI 48109‐2218 USA; ^10^ Department of Clinical Laboratory Science and Medical Biotechnology College of Medicine National Taiwan University Taipei 10048 Taiwan; ^11^ Department of Laboratory Medicine National Taiwan University Hospital Taipei 10002 Taiwan; ^12^ Genome and Systems Biology Degree Program Academia Sinica and National Taiwan University Taipei 10617 Taiwan; ^13^ Nano Science and Technology Program Taiwan International Graduate Program Academia Sinica Taipei 11529 Taiwan; ^14^ Department of Chemistry National Taiwan University Taipei 10617 Taiwan

**Keywords:** library DIA, lung cancer, microfluidics, phosphorylation, single‐cell phosphoproteomics

## Abstract

Protein phosphorylation plays a crucial role in regulating disease phenotypes and serves as a key target for drug development. Mapping nanoscale‐to‐single‐cell samples can unravel the heterogeneity of cellular signaling events. However, it remains a formidable analytical challenge due to the low detectability, abundance, and stoichiometry of phosphorylation sites. Here, we present a Chip‐DIA strategy, integrating a microfluidic‐based phosphoproteomic chip (iPhosChip) with data‐independent acquisition mass spectrometry (DIA‐MS) for ultrasensitive nanoscale‐to‐single‐cell phosphoproteomic profiling. The iPhosChip operates as an all‐in‐one station that accommodates both quantifiable cell capture/imaging and the entire phosphoproteomic workflow in a highly streamlined and multiplexed manner. Coupled with a sample size‐comparable library‐based DIA‐MS strategy, Chip‐DIA achieved ultra‐high sensitivity, detecting 1076±158 to 15869±1898 phosphopeptides from 10±0 to 1013±4 cells, and revealed the first single‐cell phosphoproteomic landscape comprising druggable sites and basal phosphorylation‐mediated networks in lung cancer. Notably, the sensitivity and coverage enabled the illumination of heterogeneous cytoskeleton remodeling and cytokeratin signatures in patient‐derived cells resistant to third‐generation EGFR therapy, stratifying mixed‐lineage adenocarcinoma‐squamous cell carcinoma subtypes, and identifying alternative targeted therapy for late‐stage patients. With flexibility in module design and functionalization, Chip‐DIA can be adapted to other PTM‐omics to explore dysregulated PTM landscapes, thereby guiding therapeutic strategies toward precision oncology.

## Graphical abstract

The integrated phosphoproteomic chip (iPhosChip) platform has paved the way for the first nanoscale‐to‐single cell phosphoproteomic analysis. The iPhosChip functions as an all‐in‐one station that hosts both quantifiable cell capture/imaging and the entire phosphoproteomic workflow from cell lysis to enriched phosphopeptides in a highly streamlined and multiplexed manner.

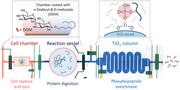

## 1 Introduction

Protein phosphorylation drives various aspects of cellular processes, such as intracellular signaling and intercellular communication.^[^ ^1^, ^2^ ^]^ To date, such signal transduction information is largely acquired through ensemble‐level measurements, which present limitations in unraveling the intricacies of signal flux across cellular networks and obscure the underlying heterogeneity. Conventional technologies, such as immunocytochemistry, flow cytometry, and mass cytometry, enable the detection of phosphoproteins in individual cells using affinity‐based reporters.^[^ ^3^, ^4^ ^]^ However, these methods require highly specific antibodies for site‐specific phosphorylation and have limited multiplexity for exploring unknown phosphorylation events. Alternatively, recent progress in mass spectrometry (MS)‐based single‐cell proteomics (SCP) methods has showcased excellent sensitivity, illuminating the individual proteome landscape with unprecedented detail and precision.^[^ ^5^, ^6^, ^7^ ^]^ Single‐cell proteomic technologies, including the nanoliter‐scale oil‐air‐droplet (OAD) chip, nanoPOTS (Nanodroplet Processing in One Pot for Trace Samples), integrated proteome analysis device for single‐cell analysis (iPAD‐1), digital microfluidic isolation of single cells for‐omics (DISCO), digital microfluidic (DMF‐SP3) chip, and nested nanowell chip (N2), as well as our previously developed microfluidic‐based platform, SciProChip, were primarily designed for processing proteomic sample preparation.^[^ ^8^, ^9^, ^10^, ^11^, ^12^, ^13^, ^14^ ^]^ However, these SCP technologies do not address post‐translational modification (PTM) analyses, such as protein phosphorylation, which is substantially more challenging. Mapping nanoscale‐to‐single‐cell phosphoproteomics remains a formidable analytical challenge due to technical hurdles stemming from the low abundance of phosphopeptide species (<1% of the total proteome), the requirement for milligram‐to‐microgram samples (>5000 cells), complexity of the sample processing protocols, and limited sensitivity of conventional phosphoproteomic workflows.^[^ ^5^, ^15^ ^]^ Ultrasensitive approaches remain to be established to delineate the signaling events and intercellular communications that are critically associated with abnormal cellular physiological states and disease phenotypes in rare cell subpopulations.

Substantial efforts have been made to improve the sensitivity of MS‐based phosphoproteomic analyses. For sample input at the microscale (µg) level, pioneering works managed to achieve a coverage of 995 phosphopeptides from 10000 HeLa cells (2 µg) using a miniaturized sample workflow,^[^ ^16^ ^]^ and 300 phosphopeptides from 0.1 µg HeLa digest (≈500 cells) using an automated enrichment protocol with TiO_2_ and Fe(III)‐IMAC cartridges.^[^ ^17^ ^]^ Similarly, a streamlined EasyPhos platform in a 96‐well plate achieved ≈8000 phosphopeptides from 12.5 µg (≈6 × 10^4^ cells) lysate.^[^ ^18^, ^19^ ^]^ Recently, a tandem tip‐based workflow was reported to enhance sensitivity to ≈3000 phosphopeptides from 1 µg (≈5000 cells) lysate.^[^ ^20^ ^]^ Through informatics mining of hundreds of single‐cell proteomic datasets, ≈60 phosphopeptides were identified across a total of 443 single cells.^[^ ^21^ ^]^ Despite these efforts, measuring the phosphoproteome at the level of a few hundred cells, or down to the single‐cell level, still requires significant innovation to advance sample processing efficiency for mapping signal transduction networks for biomedical applications. The gap in sensitivity between proteomics and phosphoproteomics is evident from a comparative summary of low‐input (including single‐cell) proteomics and phosphoproteomics (Figure S1, Supporting Information). While advancements in proteomic‐based platforms have enabled the identification of 3000–4000 proteins per cell, the best phosphoproteomic profiling to date has reported the identification of 200–600 phosphopeptides from 100 cells (boosted with 1 µg lysate, with a total input amount of ≈5100 cells).^[^ ^20^ ^]^

For ultrasensitive analytical devices, microfluidic technologies have become attractive platforms due to their inherent features, such as flexible design and integration, versatile surface functionalization, multiplexing capacity, and the ability to handle samples at the nanoliter scale.^[^ ^8^, ^9^ ^]^ In particular, microfluidic devices based on multilayer soft lithography offer excellent design flexibility for integrating various modules for sample manipulation and analysis.^[^ ^22^ ^]^ For instance, in our previous work, we developed an integrated proteomic chip (SciProChip) that successfully integrated various modules to achieve a streamlined workflow for SCP analysis with superior sensitivity and reproducibility.^[^ ^10^ ^]^ Despite the broad implementation of microfluidics in various applications, its use for PTMs analysis is lacking, likely due to the challenges associated with chip design and surface modification required to incorporate complex multistep workflows with minimal sample losses, nanoliter volume processing, enrichment modules, and surface reagent incompatibilities.

In this study, we report a novel microfluidic chip integrated with data‐independent acquisition (DIA) MS, termed “Chip‐DIA” strategy, to enable highly sensitive nanoscale‐to‐single‐cell phosphoproteomic characterizations. The protein content from 1000 to 10 cells is approximately in the range of nanograms (200–2 ng), while typical single cells contain ≈200 pg of protein. Therefore, we used the term “nanoscale” to represent analysis of small cell inputs (1000–10 cells) using Chip‐DIA, which ultimately enabled true single‐cell profiling. Specifically, an integrated phosphoproteomic chip (iPhosChip) was designed and chemically functionalized as an automated, all‐in‐one station encompassing multiplexed and quantifiable cell capture/imaging, cell lysis, protein digestion, and phosphopeptide enrichment without the need for pre‐ and post‐enrichment desalting steps. In addition to novel chip fabrication, the phosphoproteomics protocol was specifically optimized to incorporate non‐ionic and ionic detergents under controlled pH and acidic conditions, achieving: 1) a substantially simplified phosphoproteomic workflow compatible with polydimethylsiloxane (PDMS) chips with minimal surface adsorption, and 2) enhanced phosphopeptide enrichment specificity through a miniaturized TiO_2_ packed column. Furthermore, by adapting sample size‐comparable spectral libraries,^[^ ^23^ ^]^ Chip‐DIA offered superior phosphopeptide mapping coverage, detecting 15869±1898, 11962±638, 5972±438, 2292±1010, 1076±158, and 193±32 phosphopeptides from nanogram samples (1000–10 cells) down to a single‐cell, respectively. To the best of our knowledge, this is the first nanoscale‐to‐single‐cell phosphoproteomic study. The feasibility of Chip‐DIA was further demonstrated by using patient‐derived non‐small cell lung cancer (NSCLC) cells to identify potential therapeutic vulnerabilities and suggest drug options for patients with late‐stage NSCLC after developing resistance to third‐generation epidermal growth factor receptor (EGFR) therapy.

## 2 Results and Discussion

### 2.1 Design of iPhosChip as a Streamlined Phosphoproteomics Station

**Figure 1:**
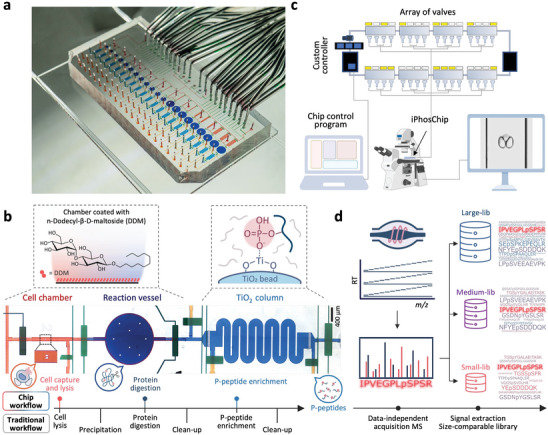
Schematics of the integrated phosphoproteomic chip (iPhosChip) and streamlined phosphoproteomic workflow for nanoscale‐to‐single‐cell phosphoproteomic analysis. a) A bright‐field image of the iPhosChip, showcasing its functional modules for multiplexed cell capture, counting, imaging, and sample processing. b) A close‐up view of a single operation unit, featuring a cell chamber (red), a reaction vessel (deep blue), and a TiO_2_ column for phosphopeptide enrichment (light blue). Control layers are shown in green. The upper left image depicts the selective coating of the cell and digestion chambers with 0.01% n‐Dodecyl‐β‐D‐maltoside, while the upper right illustrates the phosphopeptide enrichment process using packed TiO_2_ beads. The bottom portion illustrates the operation procedures of iPhosChip for streamlined sample preparation, including (1) cell trapping, imaging, and counting; (2) cell lysis; (3) protein digestion; and (4) phosphopeptide enrichment, eliminating the need for conventional desalting steps. c) A schematic diagram depicting the setup of the control system, which includes a series of pneumatic valves, the iPhosChip mounted on a microscope for real‐time monitoring of the entire sample processing workflow, and a chip control program. d) Phosphoproteomic analysis using data‐independent acquisition mass spectrometry coupled with sample size‐comparable spectral library to enhance identification coverage. Some parts of the figure were created with BioRender.com.

Designing a miniaturized phosphoproteomic sample processing platform is challenging due to the complex and lengthy multistep workflow, which makes it difficult to configure into one‐pot‐like miniaturized devices for sensitive phosphoproteomic profiling of low‐cell input samples. To realize a streamlined and sensitive workflow for phosphoproteomics analysis from the nanoscale‐to‐single‐cell samples, we leveraged our recent experience in developing the SciProChip for SCP profiling.^[^ ^10^ ^]^ Compared to SCP analysis, iPhosChip has been substantially redesigned with key features to account for the more complex phosphoproteomic workflow. This includes the use of various diverse reagents, chemical functionalization, bypassing the need for pre‐ and post‐enrichment desalting steps, and incorporation of a miniaturized TiO_2_ column for phosphopeptide enrichment (**Figure** **1**a; Figure S2a, Supporting Information). Additionally, microfluidic modules offering all the functionalities for the proteomic workflow, including quantifiable cell capture and imaging, efficient protein extraction, and digestion, have been modified to improve the overall assay performance (Figure 1b). The narrow‐body cell‐capturing chambers, patterned with a twin‐pillar array of 5 µm‐spacing, were designed to efficiently trap 1, 10, 50, 100, 500, and 1000 cells in triplicate channels.^[^ ^5^, ^6^, ^24^, ^25^ ^]^ Circular digestion vessels were tailored to accommodate sample size‐dependent processing volumes ranging from 130 to 530 nL for 1–1000 cells. Adjacent to each digestion vessel, a serpentine column with a cross‐section of 200 µm × 25 µm (width × height) was fabricated and packed with 10 µm TiO_2_ microbeads to perform phosphopeptide enrichment. For each column, the inlet and outlet channels were adjoined to enable accurate fluid operation to activate TiO_2_ microbeads, and enrich phosphopeptides and subsequently elute the bound phosphopeptides (Figure S2b, Supporting Information). The entire iPhosChip sample processing was precisely controlled by a custom controller containing 36 microvalves via a built‐in control layer. The system was actuated by sinusoidal valve arrays and guided by a graphic user interface to enable automated and parallelized operation (Figure 1c).^[^ ^10^, ^26^ ^]^

To implement the microfluidic chip for streamlined phosphoproteomic processing, we addressed technical hurdles related to reagent‐chip incompatibility and workflow complexity. By carefully selecting PDMS‐compatible reagents, we developed a simplified workflow with three key components (Figure 1b; Figure S3, Supporting Information). First, the chip was thoroughly coated with 0.01% n‐Dodecyl‐β‐D‐maltoside (DDM), a non‐ionic detergent that binds more strongly to hydrophobic surfaces than peptides and is compatible with LC‐MS/MS, to minimize non‐specific sample adsorption.^[^ ^27^ ^]^ Second, a sodium deoxycholate (SDC)‐based protocol was designed for one‐step lysis, reduction, and alkylation, which facilitates direct protein trypsinization (2 h), and phosphopeptide enrichment without the need for both pre‐and post‐enrichment desalting steps. Third, on‐chip phosphopeptide enrichment was achieved using a miniaturized TiO_2_ column with an enrichment buffer containing trifluoroacetic acid (TFA) and lactic acid to improve recovery and specificity by preventing competitive binding from acidic peptides. Compared to conventional phosphoproteomic protocols, this miniaturized chip‐based protocol streamlines and simplifies the entire workflow, minimizes sample transfer steps, and reduces sample loss. These improvements enhance sample recovery, enabling the identification of a greater number of phosphopeptides with higher sensitivity, even from minimal sample inputs. The overall sample processing time in the iPhosChip was 7 h in a multiplexed processing manner, compared to the 24 h required for conventional workflows. The operation time in iPhosChip is shortened via reduced digestion time compared to conventional overnight digestion. Moreover, this prevents evaporation‐associated sample losses, particularly for low‐input samples, allowing the completion of the entire workflow in a relatively shorter duration with minimal losses.

We recently reported a hybrid phosphopeptide spectral library that enables deep tissue coverage of more than 35000 phosphosites using single‐shot DIA workflow.^[^ ^28^ ^]^ Interestingly, at low sample inputs, we found that small spectral libraries constructed from comparable sample sizes significantly enhanced proteome coverage for mapping low‐abundance proteins from nanoscale samples.^[^ ^23^ ^]^ To further boost profiling coverage for nanoscale‐to‐single‐cell samples, we established several spectral libraries of comparable sample sizes to enable in‐depth library‐based DIA profiling of mass‐limited samples (Figure 1d).

### 2.2 Performance of iPhosChip‐Based Phosphoproteomics Workflow

**Figure 2:**
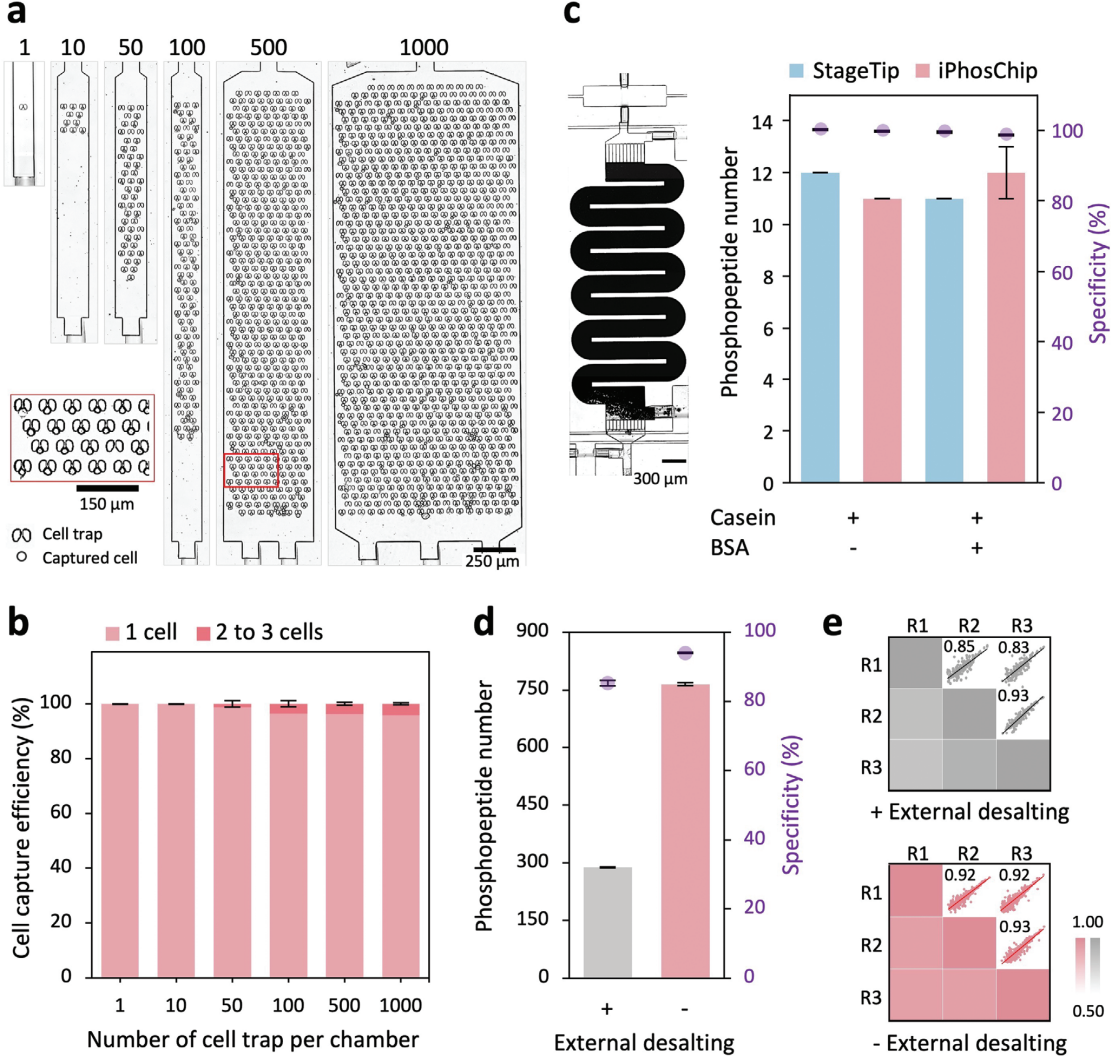
Evaluation of the iPhosChip‐based phosphoproteomic sample processing protocol. a) Brightfield images of PC9 cells captured by 1, 10, 50, 100, 500, and 1000 cell chambers. Bottom‐left: a zoom‐in image of cells captured in the 500‐cell chamber. b) Evaluation of PC9 cell capture efficiency per chamber for 1 to 1000 cell chambers (*n* = 3 independent experiments). c) The left image displays the phosphopeptide enrichment micro‐column packed with 10 µm TiO_2_ beads. The right image shows the comparison of phosphopeptide enrichment efficiency between on‐chip (rose) and conventional TiO₂‐packed StageTip (sky blue) using a mixture of phosphoprotein casein and control BSA (1:100 ratio). It shows summary of identified phosphopeptides and enrichment specificity from samples with or without BSA (*n* = 3 independent experiments). Comparison of the (d) number of phosphopeptides and (e) reproducibility of quantified phosphopeptides using the optimized miniPhos workflow for phosphoproteomic profiling of 50 PC9 cells, with and without the external desalting step (*n* = 3 independent cell samples for each condition). All the data are shown as mean ± SD from triplicate analyses.

The performance of the iPhosChip was systematically assessed. First, we evaluated cell capture efficiency by injecting PC9 cells (125 cells µL^−1^) into the cell chambers. Across 1–1000 cell chambers, the average percentage of singly‐captured cells ranged between 95.8 ± 4.2% and 100 ± 0.0%, demonstrating the high capture efficiency of the iPhosChip (**Figure** **2**a,b). Meanwhile, cell usage efficiency, defined as the percentage of trapped cells among the total injected cells, was found to span from 33.3 ± 0.0 to 72.3 ± 2.1%. Notably, although the 10‐cell chamber showed a lower efficiency of 11.3 ± 5.4%, it still showed a substantial improvement compared to ≈4% observed with the previous iProChip (Figure S4a, Supporting Information).^[^ ^10^ ^]^ Meanwhile, the higher‐viscosity reagents and different digestion vessel sizes in the current workflow were expected to affect the mixing process. Thus, the mixing efficiency was characterized. Briefly, the enrichment reagents were colored (mixed) with food dyes for visualization, dispensed into vessels, shaken (via a plate shaker), and time‐lapse images were taken. The analysis showed that complete mixing was achieved by 60 min (Figure S4b, Supporting Information).^[^ ^29^ ^]^ Next, the enrichment efficiency of TiO_2_‐packed microcolumn was evaluated using standard phosphoproteins α/β‐casein in the presence of BSA (1:100 ratio). The on‐chip strategy successfully enriched 12 ± 1 phosphopeptides with good enrichment specificity (98.8 ± 0.0%), demonstrating an enrichment capability comparable to that of the StageTip (Figure 2c).

We then applied this miniaturized phosphoproteomic (miniPhos) workflow to process 50 PC9 cells using the iPhosChip and compared it to the conventional method, which includes an external StageTip desalting step.^[^ ^16^, ^19^ ^]^ Notably, miniPhos approach identified 765 ± 4 phosphopeptides with 94% specificity from 50 cells, whereas the conventional method identified 288 ± 1 phosphopeptides with 85% specificity (Figure 2d). The miniPhos‐in‐iPhosChip approach covered 96% of the phosphopeptides identified by the conventional workflow, with 2.6‐fold more phosphopeptides identified (Figure S5a, Supporting Information). These phosphopeptides, which were uniquely observed in the miniPhos approach, were likely lost during desalting or sample transfer steps in the conventional method (Figure S5b, Supporting Information). Additionally, the elimination of both pre‐and post‐enrichment desalting steps in iPhosChip slightly improved reproducibility (Pearson's correlation coefficient r ranged from 0.92 to 0.93) in comparison to the conventional method (r = 0.83–0.93) (Figure 2e). We also assessed the effect of surface coating on the iPhosChip device, which has been previously demonstrated to reduce surface adsorption for low‐input samples.^[^ ^27^, ^30^ ^]^ Surface coating with DDM substantially increased the number of identified phosphopeptides from 343 to 733 in samples from 50 PC9 cells, covering 95% of the peptides detected with the uncoated chip (Figure S6a, Supporting Information). Consistently, the intensity of the commonly detected peptides was slightly higher in the DDM‐coated chip compared to the uncoated chip (Figure S6b, Supporting Information). Moreover, the uniquely identified phosphopeptides from the DDM‐coated chip were slightly longer (Figure S6c, Supporting Information), which likely made them prone to adsorptive loss, contributing to overall sample loss and reduced sensitivity. Taken together, these data suggest that the miniPhos protocol, with minimal processing steps, is feasible for phosphoproteomic profiling of small numbers of cells previously deemed inaccessible.

### 2.3 Phosphoproteomic Profiling of 1000–10 Cells by Chip‐DIA

**Figure 3:**
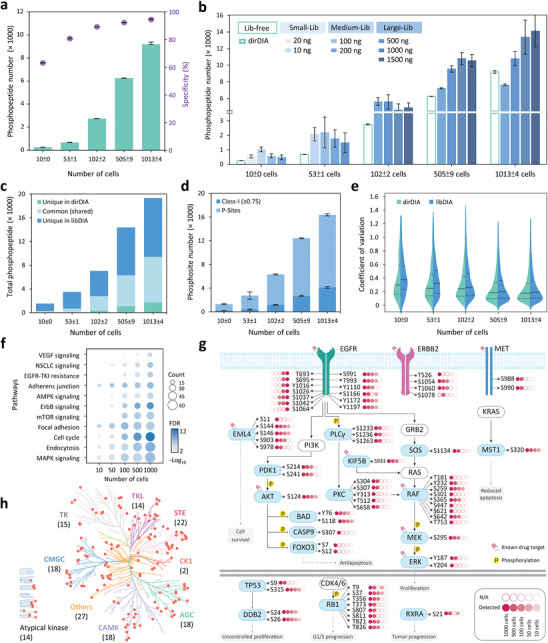
Performance evaluation of the Chip‐DIA strategy in phosphoproteomic coverage, reproducibility, and quantitation for 10–1000 PC9 cells. a) Number of unique phosphopeptides identified by triplicate analyses using directDIA (dirDIA, Spectronaut 17.2) from 10 to 1000 cells processed by Chip‐DIA (*n* = 3 independent cell samples for each condition). b) Library‐based DIA (libDIA) analysis increased the number of unique phosphopeptides identified in samples with different cell numbers. This figure illustrates the effect of different sizes of spectral libraries, which were constructed from DIA datasets of different lysate/cell quantities (1500, 1000, and 500 ng PC9 lysate and 1000 (200 ng), 500 (100 ng), 100 (20 ng), and 50 (10 ng) PC9 cells), on phosphopeptide identification in low‐input cells (*n* = 3 independent cell samples for each condition). c) Total number of phosphopeptides identified by combining dirDIA and libDIA results from 10 to 1000 cells. d) Average number of phosphorylation sites and confidently localized phosphorylation sites (class‐1; localization probability ≥75) identified from 10 to 1000 cells (*n* = 3 independent cell samples for each condition). e) Violin plot showing the distribution of coefficient of variation (CV%) of quantified phosphopeptide intensities by dirDIA and libDIA results (*n* = 3 independent measurements). The median is indicated as a solid line while quartiles are shown using dotted lines. f) Pathway enrichment analysis of phosphoproteins across 1000–10 cells using the KEGG database. The bubble size is proportional to the number of observed phosphoproteins in enriched pathways. The bubble color represents the false discovery rate (FDR) of enriched pathways. g) Mapping coverage of phosphosites and phosphoproteins in the NSCLC pathway under different cell inputs. h) Kinase tree mapping against human kinome database revealed 148 kinases from 1000 cells, with the number of kinases per family indicated in brackets. All the data are shown as mean ± SD from triplicate analyses.

We first evaluated the iPhosChip performance across a range of 1000–10 cells. By a library‐free approach (dirDIA) using directDIA+ in Spectronaut,^[^ ^31^ ^]^ an average of 9203 ± 182, 6252 ± 15, 2747 ± 28, 680 ± 1, and 253 ± 5 phosphopeptides were identified from 1013 ± 4, 505 ± 9, 102 ± 2, 53 ± 1, and 10 ± 0 PC9 cells, respectively (**Figure** **3**a; Data Set S1, Supporting Information). Notably, Chip‐DIA demonstrated high enrichment specificities of 95%, 92%, and 89% for 1000–100 cells, which slightly reduced to 81% and 63% in 50 and 10 cells, respectively (Figure 3a). It is noted that directDIA+ (deep) in Spectronaut v17 leverages improved machine learning algorithms, leading to significant 2.2–3.0‐fold increase in identification coverage compared to the classic directDIA in previous versions (Figure S7a,b, Supporting Information).

Compared to bulk samples, low‐input samples yield lower numbers and abundances of peptide mixtures, affecting the DIA‐MS/MS fragmentation patterns for subsequent deconvolution and spectral matching in the spectral library.^[^ ^23^ ^]^ To address this, we proposed a cell number‐comparable library‐based DIA (libDIA) to enable deep phosphoproteome profiling.^[^ ^23^ ^]^ Using DIA dataset from PC9 cells, we constructed spectral libraries of different sizes to evaluate their impact on the identification results. In particular, seven spectral libraries were constructed, including large‐size libraries (1500 ng/≈7500 cells; 1000 ng/≈5000 cells; 500 ng/≈2500 cells), medium‐size libraries (200 ng/≈1000 cells; 100 ng/≈500 cells), and small‐size libraries (20 ng/≈100 cells; 10 ng/≈50 cells), which yielded depths of 34174–17060, 8725–6893, and 3027–775 phosphopeptides, respectively (Table S1, Supporting Information). The library size significantly impacted phosphopeptide coverage across all datasets (Figure 3b; Data Set S2, Supporting Information). At higher cell inputs, 1000‐ and 500‐cell datasets showed best mapping results when using the large‐scale libraries (1500 ng/≈7500 cells; 1000 ng/≈5000 cells) as reflected in their higher coverage, while 100‐to‐10‐cell datasets achieved optimal results with medium and small‐scale libraries. For example, in the 10‐cell dataset, the libDIA search using a small library (20 ng/≈100 cells) achieved the highest coverage (1027 phosphopeptides), outperforming the searches against larger libraries (480–579 phosphopeptides), likely due to large query space and less comparable spectral similarity.^[^ ^23^ ^]^ Compared to dirDIA, libDIA with the best‐performing libraries significantly enhanced identification by 1.5, 1.7, 2.0, 3.2, and 4.0‐fold for 1000, 500, 100, 50, and 10 cells, respectively. By integrating dirDIA and libDIA results, we identified a total of 19323, 14367, 7037, 3486, and 1538 unique phosphopeptides (Figure 3c), corresponding to averages of 16359 ± 318, 12420 ± 127, 6330 ± 136, 2742 ± 698, and 1313 ± 100 phosphosites in 1000, 500, 100, 50, and 10 cells, respectively (Figure 3d). These findings highlight that scaling the spectral library size comparable to that of the input sample substantially enhances phosphoproteomic coverage, particularly for low‐input samples. As anticipated, smaller cell samples exhibited fewer confidently localized class‐1 sites (probability ≥ 0.75) (Figure S6b, Supporting Information), likely due to the enhanced detection of low‐abundance phosphopeptides by libDIA. Nevertheless, libDIA still demonstrated higher sensitivity in detecting more class‐1 sites compared to dirDIA.

Next, we assessed the reproducibility of Chip‐DIA through triplicate analyses across 1000–10 cells. The results revealed that 66% of phosphopeptides and 80% of phosphoproteins were commonly identified across the triplicates for 100–1000 cells, with reduced overlap observed in lower‐input cell numbers (Figure S8, Supporting Information). As expected, the majority of phosphopeptides identified from low‐input samples overlapped with those identified in high‐input samples (Figure S9a, Supporting Information). The median coefficient of variation (CV) between replicates was 17–29% for dirDIA and slightly higher (18–37%) for libDIA across all cell inputs (Figure 3e), likely due to the quantification of low‐abundance peptides by Spectronaut. Furthermore, pairwise analysis of phosphopeptide intensities across replicates revealed Pearson's correlation coefficients of 0.9 across 10–1000 cells (Figure S9b, Supporting Information), demonstrating high reproducibility. In summary, Chip‐DIA enabled reproducible nanoscale phosphoproteomic profiling with unprecedented sensitivity.^[^ ^16^, ^20^, ^32^ ^]^

Next, we evaluated whether Chip‐DIA results from 10 to 1000 cells offered sufficient coverage depth to explore lung cancer biology. Pathway analysis against Kyoto Encyclopedia of Genes and Genomes (KEGG) database revealed enrichment of top‐ranking lung cancer‐related pathways, including mitogen‐activated protein kinase (MAPK) signaling, actin cytoskeleton, ERBB signaling, non‐small cell lung cancer (NSCLC) signaling, and epidermal growth factor receptor tyrosine kinase inhibitors (EGFR‐TKI) resistance pathways (*p* < 0.05, Figure 3f). Focusing on the NSCLC pathway, the major activation route for therapy, a total of 69 phosphosites from 21 phosphoproteins (Data Set S3, Supporting Information) were covered across different cell numbers. Importantly, the results mapped 9 FDA‐approved drug targets and several known activation sites, such as the autophosphorylation sites (Y1197 and Y1172) on EGFR,^[^ ^28^ ^]^ the constitutive activation site (S124) on AKT,^[^ ^33^ ^]^ and the regulatory binding site S259 on RAF,^[^ ^34^ ^]^ in as few as 50 cells (Figure 3g). By mapping the human kinome,^[^ ^35^ ^]^ 148 protein kinases were identified in 1000 cells across all the major branches of the kinase phylogenetic tree (Figure 3h). These results demonstrate the capability of Chip‐DIA in mapping a wide range of phosphorylation‐mediated signaling events, even in low cell numbers.

### 2.4 Ultrasensitive Phosphoproteomics at Single‐Cell Resolution

**Figure 4:**
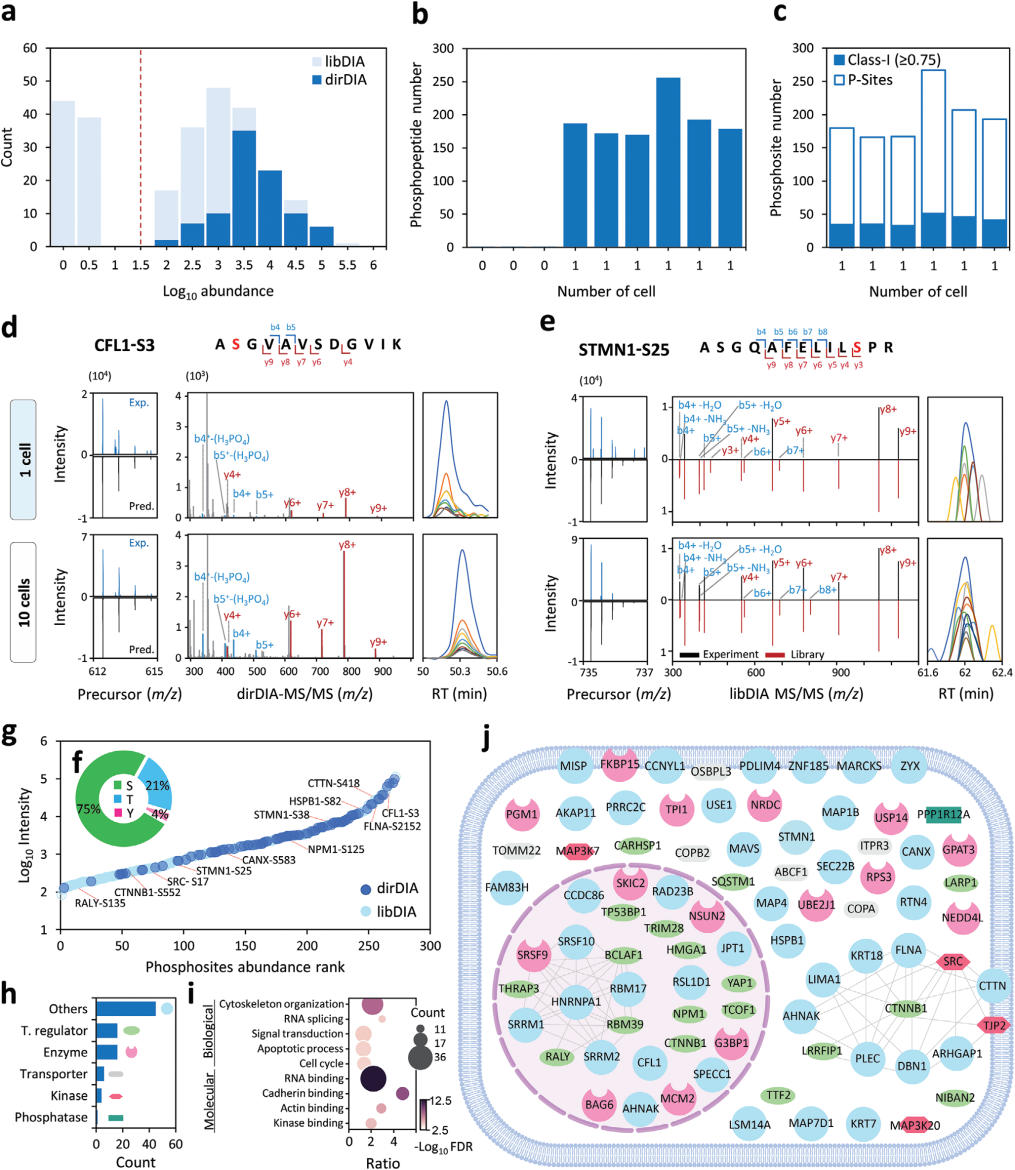
Characteristics of phosphopeptides identified in single cells by Chip‐DIA. a) Distribution patterns of phosphopeptides quantified by libDIA compared to dirDIA (*n* = 3 independent cell samples for each condition). The libDIA shows different distribution patterns of phosphopeptides toward the low‐intensity region. The red dotted line indicates the additional intensity threshold applied in the single‐cell datasets to filter out the low‐intensity phosphopeptides from the libDIA results (1% FDR precursor/peptide, and protein) to enhance confidence in identifications. b) Summary of unique phosphopeptides identified in each replicate analysis from blank samples (0 cell) and single‐cell loadings by Chip‐DIA. c) Summary of identified and localized phosphorylation sites (class‐1; localization probability ≥75) in each single cell. d) Example of a phosphopeptide commonly identified by dirDIA in both PC9 single‐cell (top) and 10 cells (bottom), along with comparison on the similarity of their raw precursor monoisotopic pattern distribution, MS/MS fragmentation pattern, and extracted ion chromatogram. e) Example of a phosphopeptide uniquely identified by libDIA in both PC9 single‐cell (top) and 10 cells (bottom), along with comparison on the similarity of their raw precursor monoisotopic pattern distribution, relative fragmentation pattern between the sample spectrum and mapped library spectrum, and extracted ion chromatogram. f) Distribution of phosphorylated serine (S), threonine (T), and tyrosine (Y) peptides detected in single cells. g) Assessment of dynamic range based on phosphosite abundance rank and annotation of proteins involved in lung cancer. h) Summary of functional categories of the identified phosphoproteins defined by Ingenuity Pathway Analysis analysis. i) Gene ontology analysis for biological processes and molecular functions using the STRING database (FDR < 0.05). j) Distribution of phosphorylated proteins based on annotated protein functional category, subcellular localization in PC9 single cells, and interaction network of cadherin binding and RNA splicing.

Encouraged by the promising results from nanoscale samples, we further evaluated the sensitivity of Chip‐DIA at single‐cell resolution. Compared to nanoscale samples, individual single‐cell samples produce significantly lower precursor and fragment signals, posing challenges for confident identification by peptide spectrum matching. At the single‐cell level, phosphopeptide precursor abundances identified by libDIA exhibited a bimodal distribution pattern, including a normal distribution mapping of more low‐abundance peptides compared to dirDIA, as well as a small group at extremely low‐intensity region (**Figure** **4**a). To improve the confidence in identifications, these low‐abundance phosphopeptides were excluded. Moreover, we used two separate chips to process single‐cell samples to compare identification results. By combining dirDIA and libDIA, similar identification coverage was observed from two independently prepared chips, with an average of 193 ± 32 phosphopeptides across six single cells, while only 1 phosphopeptide was detected in the blank samples (0 cell), demonstrating minimal contamination from samples processed in iPhosChip (Figure 4b; Data Set S4, Supporting Information). As expected, the challenge of low‐abundance phosphopeptide precursors at the single‐cell level resulted in a lower percentage of confidently localized class‐1 phosphosites (40 ± 7) (Figure 4c). Next, we used FragPipe as an alternative search tool,^[^ ^36^ ^]^ to analyze single‐cell data. The hybrid approach^[^ ^36^ ^]^ implemented in FragPipe outperformed Spectronaut's dirDIA approach, though it offered less coverage compared to Spectronaut's libDIA approach (Figure S10a,b; Data Set S5, Supporting Information). The majority (>95%) of phospho‐sequences and phosphoproteins identified by FragPipe overlapped with those identified by Spectronaut, suggesting high agreement between the two search tools (Figure S10c,d, Supporting Information). In the 0‐cell samples used as a negative control, FragPipe showed no identification results (Figure S10, Supporting Information), confirming low background contamination from iPhosChip and the reliable control of FragPipe over the false discovery rate (FDR). Nonetheless, Spectronaut's performance could be attributed to the utility of deep‐learning models for identification.^[^ ^37^ ^]^

Despite Spectronaut's statistical significance, we further assessed identification stringency by additional criteria using the 10‐cell dataset, which has a relatively higher identification coverage, as a benchmark for assessment: 1) accurate precursor mass, 2) matching monoisotopic envelope of identified and predicted precursor ion patterns, 3) matching fragment ion patterns, and 4) consistent co‐elution profile of fragment ions. In the example of the CFL1_ApSGVAVSDGVIK phosphopeptide identified from single‐cell samples using dirDIA (Figure 4d), we observed consistent patterns in the identified and predicted monoisotopic envelopes, similar fragment ion patterns, and elution profiles with lower intensity compared to the 10‐cell dataset. Similarly, the STMN1_ASGQAFELILpSPR phosphopeptide uniquely identified by libDIA in the low‐abundance region, we observed few fragment ions (b4+, y5+, y8+, and y9+) with good signal‐to‐noise ratio in the single‐cell data and similar intensity pattern compared to a series of y and b ions (b4+, b5+, and y4+ to y9+) in the 10‐cell data (Figure 4e). Despite low intensities, the high correlation of precursor and DIA‐MS/MS features between 1‐ and 10‐cell datasets confirmed identification confidence and demonstrated the utilization of sample size‐comparable libraries to recover low‐abundance phosphopeptides.

To the best of our knowledge, this study presents the first single‐cell phosphoproteomic landscape. At the basal level, serine (75%) and threonine (21%) residues remained the predominant phosphorylation sites. Interestingly, a higher percentage (4%) of tyrosine phosphorylation, predominantly enriched in the cytoskeleton, transcription, and splicing‐related proteins, was detected compared to ≈1% typically observed in bulk samples^[^ ^38^ ^]^ (Figure 4f; Table S2, Supporting Information). Moreover, ranked phosphosite abundances spanned three orders of magnitude, covering reported phosphosites known to be upregulated in lung cancer (Figure 4g; Figure S11, Supporting Information), such as RALY‐S135, CFL1‐S3, STMN1‐S25/S38, FLNA‐S2152, CTTN‐S418, CTNNB1‐S552 and HSPB1‐S82.^[^ ^39^, ^40^, ^41^, ^42^, ^43^, ^44^, ^45^ ^]^ The identified phosphosites in single cells may represent the most dominant events at the basal level. Ingenuity Pathway Analysis (IPA) revealed that the detected phosphoproteins were categorized into functional categories, including transcriptional regulators (18.2%), enzymes (18.2%), receptors and ion channels (7%), and a lower percentage of kinases (5%), and phosphatases (1%) (Figure 4h). Gene ontology analysis revealed enrichment of proteins involved in cytoskeleton organization, RNA, cadherin, and actin‐binding (Figure 4i). Cellular localization by IPA revealed that most transcriptional regulator phosphoproteins were assigned to the nucleus (11), whereas most enzymes (8), kinases (3) and transporting proteins (5) were present in the cytosol (Figure 4j; Data Set S6, Supporting Information). Among the kinases, MAP and Src‐family kinases (MAP3K7 and SRC) are reported clinical drug targets that promote tumor malignancy in NSCLC.^[^ ^46^, ^47^, ^48^ ^]^ The single‐cell landscape also encompassed the well‐known NSCLC‐related MAP kinase pathway (STMN1‐S25/S38, FLNA‐S2152, HSPB1‐S82, MAP3K7‐T44, and MAP3K20‐S3), suggesting its major role in driving basal signaling activation in NSCLC. Collectively, these results reveal that such cancer‐associated signaling events are among the most abundant phosphorylation events in single cells.

### 2.5 Nanoscale Phosphoproteomics Reveals Aberrant Cytoskeleton Remodeling and Therapeutic Vulnerability in Patients with TKI‐Resistant Lung Cancer

**Figure 5:**
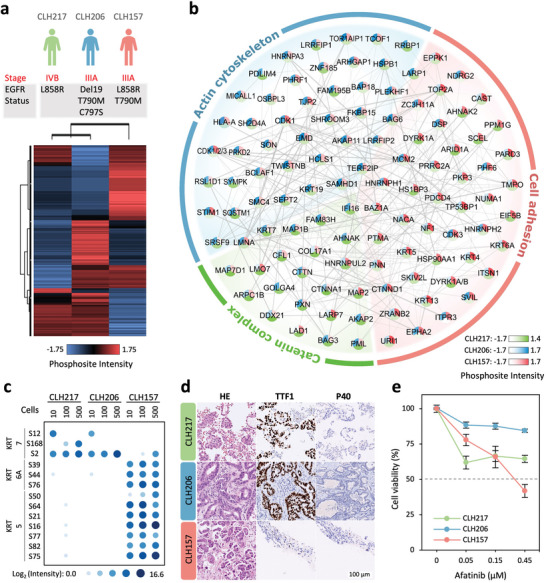
Differential phosphoproteomic profiling of lung cancer cells derived from three patients with EGFR‐TKI resistant late‐stage cancer. a) Heatmap of unsupervised clustering of 341 differentially expressed phosphosites from 10‐cell data (*n* = 3 independent cell samples for each condition)of three EGFR‐TKI resistant patient‐derived lung cancer cells (one‐way ANOVA, *p* < 0.05, s0 = 0). b) Enriched protein network and functional categories from phosphoproteins with differentially expressed phosphosites among the three cell lines, filtered with medium confidence in the STRING database (FDR < 0.05), and visualized using Cytoscape. c) Comparison of cytokeratin biomarker abundances, KRT7 (ADC subtype) and KRT5/6A (SCC subtype), in 10, 100, and 500 cells across the three cell types (*n* = 3 independent cell samples for each condition). d) Immunohistochemistry staining of hematoxylin and eosin (HE) for nuclei and cytoplasmic components, and TTF1 and P40 markers for ADC and SCC, respectively. This staining was performed to validate the tissue subtype from the surgical specimens of the three patients (scale bar = 100 µm). e) Cell viability test of three cell lines treated with indicated concentrations of afatinib, followed by a six‐day incubation period. Cell viability was assessed by AlamarBlue cell viability assay (*n* = 3 independent experiments). All the data are shown as mean ± SD from triplicate analyses.

Despite the efficacy of epidermal growth factor receptor tyrosine kinase inhibitors (EGFR‐TKIs) in prolonging the survival of NSCLC patients, unavoidable resistance to first‐ through third‐generation EGFR‐TKIs among nearly all patients remains a significant medical burden. To explore the TKI‐resistant phosphoproteome in low cell numbers and identify viable drug targets for late‐stage patients, we applied Chip‐DIA to quantitatively compare 10‐, 100‐, and 500‐cell samples from three primary lung cancer cell lines, derived from patients who acquired resistance to osimertinib (a third‐generation TKI). These cell lines included CLH157 (harboring EGFR L858R and T790M mutations), CLH206 (harboring EGFR exon 19 deletion (del19), T790M, and C797S mutations), and CLH217 (harboring L858R mutation). An average of 1475 ± 367, 1570 ± 129, and 1608 ± 104 phosphopeptides were quantified from 10 ± 1 CLH157, CLH206 and CLH217 cells, respectively (Figure S12, Supporting Information). Across all three cell lines, a total of 2083 phosphopeptides corresponding to 942 phosphorylated sequences from 590 phosphoproteins were quantified. Unsupervised clustering at the 10‐cell level revealed distinct phosphoproteomic profiles of these three cell lines (**Figure** **5**a). Differential expression analysis revealed 341 significantly altered phosphosites across the three cell lines (one‐way ANOVA, *p* < 0.05). By mapping their corresponding proteins into protein‐protein interaction networks and functional categories in the STRING database, and visualizing using Cytoscape,^[^ ^49^ ^]^ the resulting network revealed many phosphoproteins enriched in molecular events involved in cytoskeleton organization, cell adhesion, and catenin complex (Figure 5b; Data Set S7, Supporting Information]. For example, cell adhesion proteins, including EPPK1, DSP, and KRT6B showed higher phosphorylation in the CLH157 cells, while actin cytoskeleton‐associated proteins (PDLIM4, ZNF185, MICALL1) and catenin complex proteins (TNNB1, CTNNA1, CTTN) exhibited higher phosphorylation levels in CLH206 and CLH217 cells, respectively. The dramatic cytoskeleton remodeling observed in late‐stage patient‐derived cells is consistent with the reported dysregulation of cytoskeleton and associated proteins involved in tumor initiation, progression, and metastasis.^[^ ^50^, ^51^, ^52^ ^]^

Further analysis revealed that a series of cytokeratins (KRTs), essential components in the epithelial cell that play significant role in the phenotype of tumor cells,^[^ ^53^ ^]^ exhibited the most pronounced variation in phosphorylation levels (Figure 5c). Previous transcriptomics and single‐cell RNA sequencing studies have identified KRT5 and KRT6 signatures as biomarkers of squamous cell carcinoma (SCC), while adenocarcinoma (ADC) is characterized by the expression of the hallmark gene KRT7.^[^ ^54^, ^55^ ^]^ The distinct KRT5/KRT6A/KRT7 expression patterns suggest that CLH157 cells originate from SCC, while CLH206 and CLH217 cells are of ADC in origin. We further analyzed their expression pattern in 100‐ and 500‐cell samples. In CLH206 and CLH217 cells, only KRT7 phosphorylation events (S2 and S168) were observed, suggesting that both cells remain as ADC phenotype. In contrast, CLH157 cells predominantly expressed KRT5/KRT6A phosphorylation (KRT6A‐S39, S44, S76; KRT7‐S16, S21, S50, S64, S75, S77, and S82) with very low intensity of KRT7 phosphorylation detected in 100‐ and 500‐cell samples. To determine whether the SCC phenotype of CLH157 cells originated from primary lung cancer lesion (histological origin), we performed immunohistochemistry (IHC) staining for TTF1 (an ADC marker) and P40 (an SCC marker) on surgical specimens from these three patients. The IHC results revealed that tumor cells were predominantly TTF1 positive and a few P40 positive cells in CLH15, whereas only TTF1 positive tumor cells were observed in CLH206 and CLH217 (Figure 5d). Taken together, these results not only clarify why CLH206 and CLH217 cells cluster together, likely due to their shared ADC lineage, but also uncover the previously unrecognized dominance of SCC features and the lower proportion of ADC features in CLH157 cells, which may reflect the mixed‐lineage phenotype of SCC and ADC in this cell line.

Furthermore, a few drug‐responsive sites, including the autophosphorylation sites EGFR‐Y1172 and ERBB2‐S1060, were detected in CLH217 and CHL157 cells at the 100‐ and 500‐cell levels (Figure S13, Supporting Information), suggesting their potential as therapeutic targets in EGFR/ERBB2‐targeted therapies. To further explore the potential druggable opportunity, we tested the response of CLH157, CLH206, and CLH217 cells to afatinib, a known broad‐spectrum inhibitor that irreversibly blocks EGFR, ERBB2, and ERBB4 signaling,^[^ ^16^, ^19^, ^56^, ^57^ ^]^ by AlamarBlue cell viability assay. The data indicated that afatinib exhibited good to moderate inhibition of CLH157 and CLH217 cells in a dose‐dependent manner (Figure 5e). These results aligned with the findings and promise of phosphoproteomic analysis in identifying therapeutic opportunities.

Despite the strengths of this study in exploring TKI‐resistant phosphoproteome in low cell numbers, several limitations warrant consideration. First, the relatively limited coverage due to the small sample size constrains a comprehensive understanding of resistance mechanisms and potential therapeutic targets. Second, we tested only the efficacy of afatinib in inhibiting cell viability, leaving other therapeutic options unexplored and lacking clinical validation beyond cell line models. Lastly, future improvements to the iPhosChip, combined with more advanced instrumentation could enhance identification coverage, throughput, and reproducibility, potentially enabling phosphoproteomic profiling of hundreds of (single‐cell) samples.

## 3 Conclusion

In this study, we presented a streamlined Chip‐DIA strategy for ultrasensitive phosphoproteomic profiling at the nanogram to single‐cell levels. Its key features include all‐in‐one station that facilitates the streamlined operation of a conventionally complex phosphoproteomic protocol along with the adoption of sample‐size‐matched libraries to enable in‐depth and sensitive profiling of nanoscale‐to‐single‐cell samples. To date, most phosphoproteomic studies have reported minimum sample size at the microscale level (>5000–10000 cells), with coverage of ≈1000 phosphopeptides from 10000 cells and ≈3000 phosphopeptides from 1 µg cell lysate (≈5000 cell equivalents).^[^ ^16^, ^20^ ^]^ Together with sample‐size comparable libraries, Chip‐DIA offers unprecedented sensitivity, reproducibility, and coverage, identifying 15869 ± 1898 to 1076 ± 158 phosphopeptides from 1000 to 10 cells. Importantly, this approach demonstrated sensitive coverage of 193 ± 32 phosphopeptides from single cells. To the best of our knowledge, this study presents the first illumination of single‐cell phosphoproteomics landscape.

Following the demonstration of SCP for various biomedical applications, studying phosphorylation is expected to provide additional insights into biological processes and signaling cascades. At the basal level, our single‐cell phosphoproteomic data revealed phosphorylation events known to be involved in lung cancer malignancy, providing new insight that disease‐associated signaling events are among the most abundant phosphorylation events in individual cells. Additionally, Chip‐DIA revealed distinct site‐specific phosphoproteomic landscapes related to cytoskeletal remodeling, as well as differential phosphorylation patterns of cytokeratin signatures, which stratify ADC from SCC and mixed‐lineage ADC‐SCC subtype lung cancers. Importantly, the detection of phosphorylation events, such as EGFR‐Y1172 and ERBB2‐S1060, unveiled the efficacy of afatinib treatment in TKI‐resistant late‐stage patient‐derived cells. This demonstrates the enhanced sensitivity of Chip‐DIA for mapping druggable phosphosites, offering an unprecedented opportunity to guide therapeutic strategies toward precision oncology.

There are opportunities for future research to further enhance the coverage of phosphoproteomic profiling, particularly at the single‐cell level, to achieve deeper pathway mapping. The low signal of phosphate‐containing fragments and predominant neutral loss fragments present significant challenges that may require further development of dedicated FDR control strategies or specialized bioinformatics tools to improve identification confidence and site localization. Moreover, combining complementary enrichment approaches, such as other metal oxides, may enable comprehensive capture of all phosphopeptides to further improve the identification coverage.^[^ ^58^ ^]^ Compared to other microfluidic platforms, iPhosChip features enclosed chambers, making it inherently suitable for handling clinical samples that are prone to contamination. Additionally, this design also supports the incorporation of added functionalities, such as antibody functionalization to selectively capture targeted cells and interfacing with other PTM characterizations for single‐cell multi‐omics studies. In summary, Chip‐DIA is expected to hold great promise for various biomedical applications involving rare cell populations, and single cells, facilitating the exploration of cellular heterogeneity and altered signaling pathways during disease initiation and progression.

## 4 Experimental Section

### Materials and Reagents

Hexamethyldisilazane (HMDS) and chlorotrimethylsilane (TMCS) were purchased from Sigma–Aldrich (St. Louis, MO, USA). The AZ‐40XT and SU‐8 3025 photoresists and their developers were purchased from MicroChem (St. Newton, MA, USA). RTV 615 polydimethylsiloxane (PDMS) pre‐polymer and curing agent were purchased from Momentive Performance Materials (Niskayuna, NY, USA). Phosphate buffered saline (PBS; 10 mm sodium phosphate, 140 mm NaCl, pH 7.4), sodium deoxycholate (SDC), tris (2‐carboxyethyl) phosphine hydrochloride (TCEP), 2‐chloroacetamide (CAA), aqueous ammonium hydroxide, phosphatase inhibitor cocktail 2, phosphatase inhibitor cocktail 3, 2,5‐dihydroxybenzoic acid (DHB), trifluoroacetic acid (TFA), and formic acid (FA) were purchased from Sigma–Aldrich. MS grade Lysyl endopeptidase (Lys‐C) and trypsin were purchased from FUJIFILM Wako Pure Chemical Corporation (Osaka, Japan) and Promega (Madison, WI, USA), respectively. Titanium dioxide (TiO_2_) beads were purchased from GL Sciences (Cat No. 5010–75 010, Tokyo, Japan). Styrene divinylbenzene (SDB‐XC) Empore, and C8 membranes were purchased from 3M (St. Paul, MN, USA). Alamar Blue Resazurin cell viability reagent was from Thermo Fisher Scientific (Waltham, MA, USA).

### Tissue and Cell Culture

NSCLC (PC9) cell line was obtained from the RIKEN BioResource Center (Cat No. RCB4455; Ibaraki, Japan) and stocked at the institution. Cells were cultured in RPMI‐1640 medium supplemented with (10%, v/v) fetal bovine serum (FBS), sodium bicarbonate (2%, w/v), sodium pyruvate (1 mm), and 1% antibiotic‐antimycotic solution (Gibco, USA) at 37 °C in a humidified atmosphere of 5% CO_2_ and 95% air. Three primary culture lung cancer cell lines, CLH157, CLH206, and CLH217, were derived from the malignant pleural effusion of three patients receiving osimertinib as a second‐line therapy when drug resistance occurred. The CLH157 patient received 10 months of erlotinib and 4 months of osimertinib therapies. The CLH206 patient received afatinib for 20 months, osimertinib for 17 months, and osimertinib/erlotinib combination therapies for 9 months. The CLH217 patient received gefitinib, erlotinib, and Osimertinib for 4, 12, and 9 months, respectively. The CLH157 cells harbored EGFR L858R/T790M mutations but were sensitive to osimertinib. The CLH206 cells harbored EGFR exon 19 deletion/T790M/C797S mutations. The CLH217 cells only carried EGFR L858R mutation which lose EGFR T790M mutation after acquiring resistance to osimertinib. Both the CLH206 and CLH217 cells were resistant to osimertinib.

Clinical tissue samples from patients with lung cancer were obtained from the National Taiwan University Hospital. The clinical subtypes were confirmed by the pathologist. All ethical regulations have been compiled and approved by the Institutional Review Board of Biomedical Research of the National Taiwan University Hospital Research Ethics Committee (approval number: 20200311RINA). Written informed consent was obtained from all the patients.

### Design and Fabrication of iPhosChip

The iPhosChip has two layers, namely, a flow and a control layer, and was designed by the AutoCAD software (Autodesk, USA). Within the flow‐layer, there are 18 processing units, each serving to process 1–1000 cells (in triplicate). These processing units in‐turn consisted of a cell chamber, protein digestion vessel, and phosphopeptide enrichment column in concatenation. The cell chambers for 1, 10, 50, 100, 500, and 1000 have dimensions (length × width) of 941 × 100, 1677 × 215, 1677 × 215, 3691 × 215, 3330 × 621 and 3330 × 1216 µm^2^, respectively. Each cell chamber was patterned with twin cell trapping pillars in accordance with the designated cell quantity. The circular protein digestion vessels were designed to have 750, 1000, and 1,500 µm in radius for 1, 10–100, and 500–1000 processing lines, respectively. The serpentine enrichment column measured 1‐cm long and featured a cross‐sectional area of 200 µm × 25 µm. The phosphopeptide enrichment column was patterned with 23 micropillars at both ends, each with dimension of 75 × 15 µm^2^, positioned 5 µm apart. Meanwhile, the multiplexed control layer contained 36 microvalves. The corresponding photomasks for the flow and control layers were fabricated by M&R Nano Technology Co. Ltd. (Taoyuan City, Taiwan) and Taiwan Kong King Co. Ltd. (Taoyuan City, Taiwan), respectively.

Next, a regular photolithographic technique was employed to fabricate the master molds for the flow and control layers of the chip. In short, for the flow layer mold, valve structures were first fabricated using AZ 40XT photoresist, over pre‐cleaned and HMDS pre‐coated silicon wafer. To attain a height of 25 µm, AZ 40XT was span at 3500 rpm, followed by pre‐expose baking, exposure to Mercury‐match light, post‐expose baking, developing, and reflow steps. To fabricate the rest of the flow layer features, SU‐8 3025 photoresist was spun over the same wafer at 4200 rpm, to attain 25 µm height, and then span at 1800 rpm for another round to obtain additional 50 µm height, particularly at the digestion vessels. Similarly, the mold for the control layer was fabricated from SU‐8 3025 photoresist, span at 4200 rpm followed by the steps in standard fabrication protocol. Throughout the fabrication process of each mold, a mask aligner EV‐620 was used to align the layers and expose to Mercury‐match light at dosage of 250 mJ cm^−2^ through the dedicated photomasks.

To fabricate the iPhosChip as a PDMS replica of the flow and control layer molds, the wafers were pre‐treated with TMCS to avoid stickiness toward PDMS. To prepare the thicker flow layer, 60 g of the PDMS base and 6 g of the curing agent were blended using a centrifugal mixer (Thinky ARE‐310), degassed for 1 h, and poured over a pre‐treated mold. For the thin control layer, 10 g of the PDMS base and 1 g of the curing agent were blended, followed by spinning over the wafer (Laurell WS‐650 HZ‐23NPP/UD2 Spin coater). Both wafers were then baked in the oven at 80 °C for at least 45 min, followed by peeling‐off the thick flow layer from the wafer, trimming by scalpel, punching of holes (710 µm inner diameter biopsy puncher from Syneoco, USA), and cleaning using scotch tape. Then, oxygen plasma surface treatment was conducted on both flow and the control layers at the highest RF level for 1 min (Harrick plasma cleaner PDC‐001‐HP), aligned (custom stereo‐Nikon‐SMZ18 microscope with independent x, y, and z‐alignment controller), bound, and baked in oven at 80 °C overnight for hermetic seal. The bounded chip was then peeled‐off from the control layer wafer, holes were punched at the control layer, and the chips were bound again, this time onto plasma pre‐treated glass slide, and then baked at 80 °C for 48 h, making it ready for subsequent use.

### Assessment of Cell Capture and Usage Efficiency

The cell capture efficiency was assessed to determine whether the new cell chamber designs improved the single‐cell capturing and usage efficiency. First, the chambers were degassed using PBS solution to allow smooth and even flow of the cell solution. An optimized cell density (125 cells µL^−1^) was then prepared in a microtube, connected to the chip through PEEK tube (i.d = 75 µm), pressurized at 3–6 psi, and associated valves in the chip were actuated to introduce the cells into the 1–1000 cell chambers sequentially. The flow and capturing of the cells were continuously monitored in real‐time using a bright‐field microscope (Nikon‐ECLIPSE‐Ts2). Once each chamber captured a designated number of cells, the valves were closed until the next step of the workflow. To determine the minimal number of cells needed to prepare samples in the iPhosChip, PC9 cell solutions of 5, 10, and 30 µL containing 150, 300, and 900–1800 cells were prepared in separate microtubes for 1–10, 50–100, and 500–1000 chambers, respectively. The microtubes were then pressurized at 3–6 psi, connected to a degassed chip, and the appropriate valves were opened to introduce the cells into the cell chambers. Following the cell‐capturing step, the remaining volume of each microtube was measured to calculate the number of cells utilized during the process. 

### Fabrication and Characterization of Phosphopeptide Enrichment Columns on iPhosChip

The phosphopeptide enrichment columns on the iPhoschp were initially preconditioned with buffer B (80% acetonitrile (ACN), 0.1% TFA) for steady packing process. A suspension of TiO_2_ beads in buffer B (5 mg mL^−1^) was prepared in a microtube and continuously stirred using a magnetic stirrer to prevent the beads from settling. Subsequently, the microtube was connected to the bead inlet port on the chip via PEEK tube, pressurized at 12–13 psi, and the associated valves were actuated for the injection of the TiO_2_ suspension into the columns.

To evaluate the enrichment efficiency of the column, protein mixture of standard phosphoproteins (α/β‐casein) and control protein (BSA) were digested with lys‐C and trypsin at fixed enzyme to protein ratios (lys‐C, 1:100; trypsin, 1:50) at 37 °C overnight in a loBind vial. The digested peptides were desalted with in‐house prepared reverse phase SDB‐XC StageTip, and the protein concentration was determined by BCA assay following the manufacturer's protocol (Thermo Scientific). Then, spiked casein‐BSA peptides were subjected to phosphopeptide enrichment by either TiO₂ microcolumn in the iPhosChip or TiO₂‐packed StageTip‐based workflow. Meanwhile, to assess the enrichment efficiency of TiO₂ microcolumn in the iPhosChip and compare with the StageTip‐based enrichment workflow in terms of phosphopeptide identification and specificity casein peptides spiked with BSA peptides were enriched using both platforms. Briefly, the packed enrichment columns were equilibrated with buffers B and C (3.2 m lactic acid, 60% ACN, and 0.1% TFA), through infusion of each for 30 min. Next, 10 µL of casein‐BSA spiked samples (1:100 ratio) were injected through the columns from the microtube at 10 psi. Following this, buffers B and C were infused again for 1 h each to remove BSA and lactic acid residues. Finally, the enriched phosphopeptides were eluted out using 0.5% piperidine solution and analyzed by ultrafleXtreme MALDI‐TOF/TOF (Billerica, MA, USA). Briefly, the enriched casein phosphopeptide (with or without BSA) samples were reconstituted in a solution containing 50% ACN and 1% TFA. 1 µL sample was mixed with equal volume of matrix solution (20 mg mL‐1 DHB in 50% ACN with 1% H₃PO₄) and spotted onto the MALDI target plate. The dried samples were analyzed by the MALDI‐TOF. The data was acquired in positive ion linear mode from 700 to 3500 Da. Each spectrum was collected as an average of 6000 laser shots. The FlexAnalysis software (version 3.4, Bruker Daltonics, Germany) was used for raw data processing and further analysis.

### Phosphoproteomic Workflow by iPhosChip

The iPhosChip operates by connecting to the sinusoidal valves in the control system, which, are actuated by a chip‐controlling program (MATLAB software). A nitrogen gas pressure, of 28–30 psi, was used to close all the valves on the iPhosChip. In order to make the chip ready for phosphoproteomics workflow, the phosphopeptide enrichment columns were packed with TiO_2_ beads at 12 psi, and cell lysis and protein digestion chambers were coated with DDM (0.01%) to minimize the non‐specific absorption and degassed with PBS. In the first step of sample preparation, a microtube containing the cell solution (150 cells mL^−1^) was connected to the chip, and pressurized at 3–6 psi to drive the cells into the cell‐capturing chambers while being monitored in real‐time by the microscope (Figure S3, Supporting Information). In the second step, for single‐cell processing units, 15 nL of cocktail lysis buffer, composed of SDC, TCEP, and CAA, and phosphatase inhibitors (PP2 and PP3) was infused into the cell chambers for protein extraction, reduction, and alkylation. The chip was then incubated at 70 °C and shaken (350 rpm) for 30 min (Eppendorf ThermoMixer C). In the third step, 15 nL of proteolytic enzyme mixture (lys‐C and trypsin) was infused to digest the proteins for 2 h. In the fourth step, 30 nL of isopropyl alcohol was dispensed to avoid the precipitation of SDC in a lower pH environment during the phosphopeptide enrichment step. Finally, 60 nL of enrichment buffer (3.2 m lactic acid, 60% ACN, and 1% TFA) was infused for phosphopeptide enrichment. Likewise, for processing larger cell numbers (10–100 cells), injection volumes of 25, 25, 60, and 110 nL were utilized for consecutive injections of cocktail lysis buffer, proteolytic enzymes, isopropyl alcohol, and enrichment buffers, respectively, in the case of 10–100 cell processing lines. For the 500–1000 cell processing lines, injection volumes were adjusted to 65, 65, 130, and 260 nL for the same sequential injections. Next, the packed TiO_2_ beads in the enrichment columns were washed with buffer B (80% ACN, and 0.1% TFA) and equilibrated with buffer C (3.2 m lactic acid, 60% ACN, and 0.1% TFA). Then, the samples were loaded from the digestion vessels across all columns under 11 psi. It was noted the samples were pushed for 49 s and incubated for 2–3 min to enhance the interaction between the phosphopeptides and TiO_2_ beads. After that, buffer C was infused to wash out free unbound peptides, followed by infusion of buffer B to remove lactic acid from the columns. Importantly, these buffers were infused through separate channels to prevent lactic acid from remaining in the final samples. Finally, an elution buffer (0.5% NH_4_OH) was infused to elute out the phosphopeptides from the columns into DDM‐coated LoBind vials. The eluted phosphopeptides were immediately placed into Speed Vac evaporator concentrator at 40 °C until dryness. The dried phosphopeptides were reconstituted with 5 µL MS loading buffer (0.1% formic acid) spiked with indexed retention time (iRT) peptides (Biognosys, Schlieren, Switzerland), and 4.5 µL was injected into the LC‐MS/MS system for subsequent analysis.

### Phosphoproteomics Workflow for Bulk Sample

For the bulk sample as control experiment, the PC9 cells (10 cm dish, 1×10^6^) were washed three times with PBS and then lysed with 0.5 mL cocktail lysis buffer containing 1% SDC, 0.01 m TCEP, 0.04 m CAA, and phosphatase inhibitors (PP2 and PP3) in 0.1 m Tris‐HCl pH 9.0. The lysed cells (lysate) were collected with a cell scraper in a 1.5 mL LoBind vial, heated at 70 °C for 30 min and sonicated with 5 cycles of pulse and pause for 5 min at 4 °C. The lysate was then centrifuged at 16000 × g for 30 min at 4 °C and the supernatant was collected for further processing. Next, BCA assay was performed to measure the protein concentration and to obtain the desired initial concentration (≈0.5–1 mg) in the final volume. The desired protein amount (0.5–10 µg) was adjusted in a final volume of 10 µL with 0.1 m Tris‐HCl and digested with Lys‐C and trypsin at an enzyme‐to‐protein ratio of (Lys‐C, 1:20; trypsin, 1:10) under constant shaking (2000 rpm) at 37 °C for 2 h. Following enzymatic digestion, an equal volume of isopropyl alcohol was added to prevent acid‐induced precipitation of SDC in the next step. The prevention of SDC precipitation allows bypassing pre‐enrichment desalting step, thereby minimizing sample loss and processing steps. For phosphopeptide enrichment, an equal volume of enrichment buffer consisting of 3.2 m lactic acid, 60% ACN, and 1% TFA (final volume ≈40 µL) was added and mixed thoroughly. Afterward, TiO_2_ beads re‐suspended in buffer B at a concentration of 75 µg µL^−1^ were added into sample and incubated at 25^ °^C with shaking (1500 rpm) for 5 min. The phosphopeptides bound to titanium were pelted by centrifugation at 1000 × g for 30 sec and the supernatants containing free peptides were discarded. The titanium beads were re‐suspended in 50 µL enrichment wash buffer C (3.2 m lactic acid, 60% ACN, and 0.1% TFA) and transferred to a pre‐packed C8 membrane D200‐StageTip with centrifugation at 1000 × g rpm for 1 min. The beads were again washed with 50 µL enrichment buffer C to improve selectivity, followed by washing 3 times with buffer B to remove bound lactic acid and free peptides. Then, the bound phosphopeptides were eluted with 20 µL 0.5% aqueous ammonium hydroxide and centrifuged at 500 × g for 5 min. The eluted phosphopeptides were immediately placed into Speed Vac evaporator concentrator at 40 °C until dryness. The dried phosphopeptides were reconstituted in a 5 µL LC‐MS/MS loading buffer (0.1% formic acid) spiked with indexed retention time (iRT) standard peptides (Biognosys, Schlieren, Switzerland) and 4.5 µL was injected into LC‐MS/MS for analysis.

### LC‐MS/MS Analysis

All LC‐MS/MS analyses were performed on Orbitrap Eclipse Tribrid mass spectrometer (Thermo Fisher Scientific, Waltham, MA, USA) coupled with a Thermo Fisher Scientific UltiMate 3000 RSLCnano system via a nano‐electrospray source. The injected phosphopeptides were separated on a reversed‐phase CSH C18 column (Waters, Milford, MA, USA; nanoEase M/Z Peptide) of 25 cm length, 75 µm inner diameter, packed with 1.7 µm particles of 130 Å pore size at 250 nL min^−1^ using buffer A (0.1% FA in water) and buffer B (0.1% FA in ACN). The loaded peptides were separated with a gradient of 3–25% buffer B for 72 min, 25–40% buffer B for 74 min, 40–95% buffer B for 76 min, 95% buffer B for 80 min, and 1% buffer B for 90 min at a flow rate of 300 nL min^−1^.

The mass spectrometer was operated in positive mode with spray voltage set to 1675 V, RF lens frequency set to 30% and ion transfer tube was heated at 305 °C for desolvation. For DIA data acquisition, the data was acquired in DIA mode covering a precursor range of 500–1000 m z^−1^ and 50 scan events of 10 Da isolation windows were employed with 1 m z^−1^ overlap. The full scan range was set to 395–1125 m z^−1^ with advanced peak determination, and the MS1 orbitrap resolution was set to 120000, with an AGC target value of 250% and an IT of 50 ms. The filtered precursor ions were fragmented by HCD with collision energy of 30%. The MS/MS scan range was set to 145–1450 m z^−1^, fragment ions were detected in the orbitrap resolution of 50000 with an AGC target of 800% and an IT of 86 ms. All the data were acquired in profile mode, and the default charge state was set to 2.

### Construction of Phosphoproteome Spectral Libraries

The project‐specific spectral libraries of different sizes were constructed using different input samples, including 1500, 1000, 500, 200, 100, 20, and 10 ng lysate (or cell equivalents) from PC9 lung cancer cell lines and patient‐derived CLH157, CLH206, and CLH217 cell lines. Samples of 1500, 1000, and 500 ng protein amount were processed using StageTip‐based method and 1000 (200 ng), 500 (100 ng), 100 (20 ng), and 50 (10 ng) cells were processed using the iPhosChip‐based method. For library generation, the raw files were acquired by DIA mode analysis (described in the previous section and were searched against UniProt Human database (UniProt Reference Proteome, Taxonomy 9606, Proteome ID UP000005640, 20 387 entries, downloaded on September 23, 2021) using the Pulsar search engine under Spectronaut v 17.2 (Quasar) software (Biognosys, Zurich, Switzerland). The raw data files were imported into Spectronaut and processed using default settings unless otherwise stated. For the setting of library generation, the enzyme parameter was set to trypsin/P with up to two missed cleavages; cysteine carbamidomethylation was set as fixed modification and a maximum of 5 modifications, including methionine oxidation, acetylation (at protein N‐terminus) and phosphorylation of serine, threonine,‐ and tyrosine (STY) were set as variable modification. The peptide length was set to 7–52 in the search space. The best most intense fragment ions (minimum = 5 and maximum = 15) with relative intensity of 5% (default in Spectronaut 17.2 is 1%) per spectrum were included, whereas the fragment ions with less than three amino acid residues were not considered. The FDR cut‐off was set to 0.01 (1%) at the peptide spectrum match (PSM), peptide, and phosphosites localization probability. The precursors with phosphorylation modifications were finally retained in the library.

### Data Analysis

The raw files were analyzed using Spectronaut v 17.2 (Quasar) software in library‐free workflow (directDIA+) as well as in‐house built spectral libraries workflow (libDIA) with default settings unless otherwise stated. For both directDIA+ and libDIA, the identification search was performed against the UniProt human proteome database (UniProt Reference Proteome, Taxonomy 9606, Proteome ID UP000005640, 20 387 entries, downloaded on September 23, 2021) containing the iRT peptide sequences. The search parameters of Spectronaut were set as follows: trypsin/P as digestion enzyme, peptide length from 7–52, maximum missed cleavages set to 2, carbamidomethyl on cysteine as static modification, acetyl at protein N‐terminus, oxidation on methionine, phosphorylation on S/T/Y as variable modifications, and maximum modifications were set to 5. The FDR was set to 0.01 at precursor and protein levels (experiment‐wise) and 0.05 at protein level (run‐wise). Cross‐run normalization was set to automatic; interference correction was enabled for both MS1 and MS2, minimum relative fragment intensity was set to 5%, best intense fragment ions was set to minimum 5 and maximum 15 per spectrum, and decoy generation method “mutated” based on neural network (NN) predicted fragments was used. Following Spectronaut processing, the peptide and site reports for all searches were exported for further statistical and bioinformatic analyses.

For FragPipe (version 20.0) analysis,^[^ ^36^ ^]^ the raw files were first converted to mzML format using ProteoWizard.^[^ ^59^ ^]^ Next, DIA_SpecLib_Quant workflow was loaded, and the parameters were adjusted to closely align with Spectronaut. The peptide length was set from 7 to 50, the maximum allowed missed cleavages set to 2, and the maximum variable modifications on a peptide were set to 3. Carbamidomethyl on cysteine was set as fixed modification. Acetylation at protein N‐terminus, Oxidation on methionine, and Phosphorylation on S/T/Y were set as variable modifications. Single‐cell mzML files were set as DIA data type while library files (50‐cell mzML files) were set as DIA‐Lib data type. Similar settings were employed for blank samples. For the spectral library generation, the precursors were filtered with 1% global peptide and protein‐level FDR. For quantification, the precursors were filtered with 1% global and run‐specific precursor‐level FDR. Precursors without any MS1 signals were discarded.

### Immunohistochemistry Staining

Four µm‐thick tissue sections were dewaxed and rehydrated. For hematoxylin‐eosin staining, tissue sections were treated with hematoxylin solution, followed by counterstaining with eosin Y solution. For immunohistochemical staining, the tissue sections were deparaffinized using EZ prep (Roche Diagnostics, Tucson, AZ, USA) and subjected to a 64 min pre‐treatment using Ultra Cell Condition 1 solution (Roche Diagnostics). The slides were incubated with anti‐TTF1 antibody (SP141, ready‐to‐use, Roche Diagnostics) and anti‐p40 antibody (BC28, ready‐to‐use, Roche Diagnostics). The OptiView DAB Detection Kit (Roche Diagnostics) was used to detect protein expression. All sections were counterstained with hematoxylin. The IHC was performed on the Benchmark Ultra platform (Roche Diagnostics) according to the manufacturer's instructions.

### Cell Viability Assay (Alamar‐Blue Assay)

The cells were trypsinized and suspended in RPMI‐1640 medium containing 10% serum to form cell suspension. Next, cells were counted and inoculated into 96‐well plates with 3 × 10^3^ cells per well. The cells were then treated with three doses (0.05, 0.15, and 0.45 µm) of afatinib and incubated for 6 days. To evaluate cell viability, the cells were incubated with Alamar Blue (resazurin‐based solutions) for 8 h, and the absorbance was measured at 570 nm/600 nm by Wallac Victor3 1420 multilabel counter (Perkin Elmer). The cell viability at different drug concentrations was plotted for all three cell types.

### Statistics Analysis

A minimum of three technical or biological replicates was used to generate the data. For quantitative comparison of triplicate datasets from three cell lines (CLH157, CLH206, and CLH217), all statistical analyses were performed using Perseus software (1.6.15.0).^[^ ^60^ ^]^ All the phosphopeptides or phosphosites intensities from Spectronaut (log_10_ fragment intensity >1.5) were transformed using a logarithmic (log_2_) function. Only phosphosites quantified in at least two replicates from at least one cell line were filtered for further analysis. The missing values were imputed with relatively small constant values (<0.1%). One‐way analysis of variance (ANOVA) test on the phosphopeptide intensities was applied for quantitative comparison to identify differentially expressed phosphorylated sites (*p* < 0.05, s0 = 0). For the input of unsupervised clustering analysis of the three cell lines, the phosphosite intensities of each replicate were subjected to z‐score transformation, and the average of triplicate data of the differentially expressed sites were clustered using Pearson distance in the Perseus software. For network enrichment analysis, the gene names of differentially expressed phosphosites were imported into the web‐based STRING (v 11.5, https://string‐db.org)^[^ ^61^ ^]^ tool for assembly of functional networks with a minimum interaction score cutoff of 0.4 (medium confidence). The functional interaction networks built in STRING were imported into Cytoscape (v3.9.1)^[^ ^49^ ^]^ for further processing and visualization. KEGG pathway enrichment analysis was performed using DAVID (2021, http://david.ncifcrf.gov). The protein subcellular localization and protein category were annotated using the Ingenuity Pathways (IPA‐Ingenuity Systems, QIAGEN) from the list of phosphoproteins identified in single PC9 cells. All the data are shown as mean ± SD from triplicate analyses. The bar height in bar plot shows average number of three replicates. The box in each box plot encapsulates interquartile range (IQR), with the bottom and top edges representing first (Q1) and third (Q3)‐, respectively. The median was marked by a horizontal line within the box.

## Conflict of Interest

The authors declare no conflict of interest.

## Author Contributions

G.M. and S.T.G. performed the experiments and acquired and analyzed the data. C.‐S.C. and T.‐T.L. participated in sample preparation and data analysis. C.‐A.L., M.‐S.H., and C.‐C.H. cultured patient‐derived cells and performed immunohistochemistry and cell viability assay experiments. F.Y. and A.I.N. analyzed the single‐cell data using FragPipe. Y.‐J.C. and H.‐L.T. conceived and supervised the work. G.M., S.T.G., F.Y., A.I.N., S.‐L.Y., H.‐L.T., and Y.‐J.C. wrote and edited the manuscript. All authors commented and contributed to the final editing of the manuscript.

## Supporting information



Supporting Information

Supporting Information

Supporting Information

Supporting Information

Supporting Information

Supporting Information

Supporting Information

Supporting Information

Supporting Information

Supporting Information

## Data Availability

The data that support the findings of this study are openly available in JPOST and PXD045210 for ProteomeXchange at http://www.proteomexchange.org, reference number 45210.

## References

[1] W. Kolch , M. Halasz , M. Granovskaya , B. N. Kholodenko , Nat. Rev. Cancer 2015, 15, 515. 26289315 10.1038/nrc3983

[2] E. J. Needham , B. L. Parker , T. Burykin , D. E. James , S. J. Humphrey , Sci. Signal. 2019, 12. 10.1126/scisignal.aau8645 30670635

[3] M. Tognetti , A. Gabor , M. Yang , V. Cappelletti , J. Windhager , O. M. Rueda , K. Charmpi , E. Esmaeilishirazifard , A. Bruna , N. de Souza , C. Caldas , A. Beyer , P. Picotti , J. Saez‐Rodriguez , B. Bodenmiller , Cell Syst. 2021, 12, 401. 33932331 10.1016/j.cels.2021.04.002

[4] J. Glassberg , A. H. Rahman , M. Zafar , C. Cromwell , A. Punzalan , J. J. Badimon , L. Aledort , J. Immunol. Methods 2018, 453, 11. 28760671 10.1016/j.jim.2017.07.014 PMC7487207

[5] H. Xie , X. Ding , Adv. Sci. 2022, 9, e2105932. 10.1002/advs.202105932 PMC9036017 35199955

[6] S. T. Gebreyesus , G. Muneer , C.‐C. Huang , A. A. Siyal , M. Anand , Y.‐J. Chen , H.‐L. Tu , Lab Chip 2023, 23, 1726. 36811978 10.1039/d2lc01096h

[7] T. Truong , K. G. I. Webber , S. Madisyn Johnston , H. Boekweg , C. M. Lindgren , Y. Liang , A. Nydegger , X. Xie , T.‐M. Tsang , D. A. D. N. Jayatunge , J. L. Andersen , S. H. Payne , R. T. Kelly , Angew. Chem. Int. Ed. 2023, 62, e202303415. 10.1002/anie.202303415 PMC10529037 37380610

[8] Z.‐Y. Li , M. Huang , X.‐K. Wang , Y. Zhu , J.‐S. Li , C. C. L. Wong , Q. Fang , Anal. Chem. 2018, 90, 5430. 29551058 10.1021/acs.analchem.8b00661

[9] Y. Zhu , P. D. Piehowski , R. Zhao , J. Chen , Y. Shen , R. J. Moore , A. K. Shukla , V. A. Petyuk , M. Campbell‐Thompson , C. E. Mathews , R. D. Smith , W.‐J. Qian , R. T. Kelly , Nat. Commun. 2018, 9, 882. 29491378 10.1038/s41467-018-03367-w PMC5830451

[10] S. T. Gebreyesus , A. A. Siyal , R. B. Kitata , E. S.‐W. Chen , B. Enkhbayar , T. Angata , K.‐I. Lin , Y.‐J. Chen , H.‐L. Tu , Nat. Commun. 2022, 13, 37. 35013269 10.1038/s41467-021-27778-4 PMC8748772

[11] J. Leipert , A. Tholey , Lab Chip 2019, 19, 3490. 31531506 10.1039/c9lc00715f

[12] J. Lamanna , E. Y. Scott , H. S. Edwards , M. D. Chamberlain , M. D. M. Dryden , J. Peng , B. Mair , A. Lee , C. Chan , A. A. Sklavounos , A. Heffernan , F. Abbas , C. Lam , M. E. Olson , J. Moffat , A. R. Wheeler , Nat. Commun. 2020, 11, 5632. 33177493 10.1038/s41467-020-19394-5 PMC7658233

[13] J. Woo , S. M. Williams , L. M. Markillie , S. Feng , C.‐F. Tsai , V. Aguilera‐Vazquez , R. L. Sontag , R. J. Moore , D. Hu , H. S. Mehta , J. Cantlon‐Bruce , T. Liu , J. N. Adkins , R. D. Smith , G. C. Clair , L. Pasa‐Tolic , Y. Zhu , Nat. Commun. 2021, 12, 6246. 34716329 10.1038/s41467-021-26514-2 PMC8556371

[14] X. Shao , X. Wang , S. Guan , H. Lin , G. Yan , M. Gao , C. Deng , X. Zhang , Anal. Chem. 2018, 90, 14003. 30375851 10.1021/acs.analchem.8b03692

[15] T. Y. Low , M. A. Mohtar , P. Y. Lee , N. Omar , H. Zhou , M. Ye , Mass Spectrom. Rev. 2021, 40, 309. 32491218 10.1002/mas.21636

[16] T. Masuda , N. Sugiyama , M. Tomita , Y. Ishihama , Anal. Chem. 2011, 83, 7698. 21888424 10.1021/ac201093g

[17] H. Post , R. Penning , M. A. Fitzpatrick , L. B. Garrigues , W. Wu , H. D. MacGillavry , C. C. Hoogenraad , A. J. R. Heck , A. F. M. Altelaar , J. Proteome Res. 2017, 16, 728. 28107008 10.1021/acs.jproteome.6b00753

[18] S. J. Humphrey , O. Karayel , D. E. James , M. Mann , Nat. Protoc. 2018, 13, 1897. 30190555 10.1038/s41596-018-0014-9

[19] S. J. Humphrey , S. B. Azimifar , M. Mann , Nat. Biotechnol. 2015, 33, 990. 26280412 10.1038/nbt.3327

[20] C.‐F. Tsai , Y.‐T. Wang , C.‐C. Hsu , R. B. Kitata , R. K. Chu , M. Velickovic , R. Zhao , S. M. Williams , W. B. Chrisler , M. L. Jorgensen , R. J. Moore , Y. Zhu , K. D. Rodland , R. D. Smith , C. H. Wasserfall , T. Shi , T. Liu , Commun. Biol. 2023, 6, 70. 36653408 10.1038/s42003-022-04400-x PMC9849344

[21] B. C. Orsburn , Y. Yuan , N. N. Bumpus , Nat. Commun. 2022, 13, 7246. 36433961 10.1038/s41467-022-34919-w PMC9700839

[22] A. Abdulla , T. Zhang , S. Li , W. Guo , A. R. Warden , Y. Xin , N. Maboyi , J. Lou , H. Xie , X. Ding , Microsyst. Nanoeng. 2022, 8, 13. 35136652 10.1038/s41378-021-00342-2 PMC8807661

[23] A. A. Siyal , E. S.‐W. Chen , H.‐J. Chan , R. B. Kitata , J.‐C. Yang , H.‐L. Tu , Y.‐J. Chen , Anal. Chem. 2021, 93, 17003. 34904835 10.1021/acs.analchem.1c03477

[24] D. Di Carlo , N. Aghdam , L. P. Lee , Anal. Chem. 2006, 78, 4925. 16841912 10.1021/ac060541s

[25] A. M. Skelley , O. Kirak , H. Suh , R. Jaenisch , J. Voldman , Nat. Methods 2009, 6, 147. 19122668 10.1038/nmeth.1290 PMC3251011

[26] R. Gómez‐Sjöberg , A. A. Leyrat , D. M. Pirone , C. S. Chen , S. R. Quake , Anal. Chem. 2007, 79, 8557. 17953452 10.1021/ac071311w

[27] C.‐F. Tsai , P. Zhang , D. Scholten , K. Martin , Y.‐T. Wang , R. Zhao , W. B. Chrisler , D. B. Patel , M. Dou , Y. Jia , C. Reduzzi , X. Liu , R. J. Moore , K. E. Burnum‐Johnson , M.‐H. Lin , C.‐C. Hsu , J. M. Jacobs , J. Kagan , S. Srivastava , K. D. Rodland , T. Shi , Commun. Biol. 2021, 4, 265. 33649493 10.1038/s42003-021-01797-9 PMC7921383

[28] R. B. Kitata , W.‐K. Choong , C.‐F. Tsai , P.‐Y. Lin , B.‐S. Chen , Y.‐C. Chang , A. I. Nesvizhskii , T.‐Y. Sung , Y.‐J. Chen , Nat. Commun. 2021, 12, 2539. 33953186 10.1038/s41467-021-22759-z PMC8099862

[29] N. H. An Le , H. Deng , C. Devendran , N. Akhtar , X. Ma , C. Pouton , H.‐K. Chan , A. Neild , T. Alan , Lab Chip 2020, 20, 582. 31898701 10.1039/c9lc01174a

[30] G. Muneer , C.‐S. Chen , T.‐T. Lee , B.‐Y. Chen , Y.‐J. Chen , J. Proteome Res. 2024, 23, 3294. 39038167 10.1021/acs.jproteome.3c00862 PMC11301667

[31] R. Bruderer , O. M. Bernhardt , T. Gandhi , S. M. Miladinović , L.‐Y. Cheng , S. Messner , T. Ehrenberger , V. Zanotelli , Y. Butscheid , C. Escher , O. Vitek , O. Rinner , L. Reiter , Mol. Cell. Proteomics 2015, 14, 1400. 25724911 10.1074/mcp.M114.044305 PMC4424408

[32] A. Martínez‐Val , K. Fort , C. Koenig , L. Van der Hoeven , G. Franciosa , T. Moehring , Y. Ishihama , Y.‐J. Chen , A. Makarov , Y. Xuan , J. V. Olsen , Nat. Commun. 2023, 14, 3599. 37328457 10.1038/s41467-023-39347-y PMC10276052

[33] J. Joshi , P. J. Fernandez‐Marcos , A. Galvez , R. Amanchy , J. F. Linares , A. Duran , P. Pathrose , M. Leitges , M. Cañamero , M. Collado , C. Salas , M. Serrano , J. Moscat , M. T. Diaz‐Meco , EMBO J. 2008, 27, 2181. 18650932 10.1038/emboj.2008.149 PMC2519103

[34] A. S. Dhillon , S. Meikle , Z. Yazici , M. Eulitz , W. Kolch , EMBO J. 2002, 21, 64. 11782426 10.1093/emboj/21.1.64 PMC125807

[35] K. S. Metz , E. M. Deoudes , M. E. Berginski , I. Jimenez‐Ruiz , B. A. Aksoy , J. Hammerbacher , S. M. Gomez , D. H. Phanstiel , Cell Syst. 2018, 7, 347. 30172842 10.1016/j.cels.2018.07.001 PMC6366324

[36] F. Yu , G. C. Teo , A. T. Kong , K. Fröhlich , G. X. Li , V. Demichev , A. I. Nesvizhskii , Nat. Commun. 2023, 14, 4154. 37438352 10.1038/s41467-023-39869-5 PMC10338508

[37] R. Lou , Y. Cao , S. Li , X. Lang , Y. Li , Y. Zhang , W. Shui , Nat. Commun. 2023, 14, 94. 36609502 10.1038/s41467-022-35740-1 PMC9822986

[38] H. Zhou , S. Di Palma , C. Preisinger , M. Peng , A. N. Polat , A. J. R. Heck , S. Mohammed , J. Proteome Res. 2013, 12, 260. 23186163 10.1021/pr300630k

[39] A. J. F. Reyes , R. B. Kitata , M. A. C. Dela Rosa , Y.‐T. Wang , P.‐Y. Lin , P.‐C. Yang , A. Friedler , S. Yitzchaik , Y.‐J. Chen , Anal. Chim. Acta 2021, 1155, 338341. 33766317 10.1016/j.aca.2021.338341

[40] F. Lu , C. Zhong , Y. Dong , M. Wang , Q. Yang , Revista Romana de Medicina de Laborator 2022, 30, 379.

[41] L. Li , Y. Lu , P. M. Stemmer , F. Chen , Oncotarget 2015, 6, 12009. 25944616 10.18632/oncotarget.3617 PMC4494919

[42] G. Chen , H. Wang , T. G. Gharib , C.‐C. Huang , D. G. Thomas , K. A. Shedden , R. Kuick , J. M. G. Taylor , S. L. R. Kardia , D. E. Misek , T. J. Giordano , M. D. Iannettoni , M. B. Orringer , S. M. Hanash , D. G. Beer , Mol. Cell. Proteomics 2003, 2, 107. 12644570 10.1074/mcp.M200055-MCP200

[43] C.‐H. Chen , S.‐M. Chuang , M.‐F. Yang , J.‐W. Liao , S.‐L. Yu , J. J. W. Chen , Mol. Cancer Res. 2012, 10, 1319. 22912335 10.1158/1541-7786.MCR-12-0189

[44] B. Lelj‐Garolla , M. Kumano , E. Beraldi , L. Nappi , P. Rocchi , D. N. Ionescu , L. Fazli , A. Zoubeidi , M. E. Gleave , Mol. Cancer Ther. 2015, 14, 1107. 25740245 10.1158/1535-7163.MCT-14-0866

[45] Y.‐J. Chen , T. I. Roumeliotis , Y.‐H. Chang , C.‐T. Chen , C.‐L. Han , M.‐H. Lin , H.‐W. Chen , G.‐C. Chang , Y.‐L. Chang , C.‐T. Wu , M.‐W. Lin , M.‐S. Hsieh , Y.‐T. Wang , Y.‐R. Chen , I. Jonassen , F. Z. Ghavidel , Z.‐S. Lin , K.‐T. Lin , C.‐W. Chen , P.‐Y. Sheu , Y.‐J. Chen , Cell 2020, 182, 226.32649875

[46] J. C. Montero , S. Seoane , A. Ocana , A. Pandiella , Clin. Cancer Res. 2011, 17, 5546. 21670084 10.1158/1078-0432.CCR-10-2616

[47] H. Sakurai , Trends Pharmacol. Sci. 2012, 33, 522. 22795313 10.1016/j.tips.2012.06.007

[48] R. Santoro , C. Carbone , G. Piro , P. J. Chiao , D. Melisi , Drug Resist. Updat. 2017, 33–35, 36. 10.1016/j.drup.2017.10.004 29145973

[49] P. Shannon , A. Markiel , O. Ozier , N. S. Baliga , J. T. Wang , D. Ramage , N. Amin , B. Schwikowski , T. Ideker , Genome Res. 2003, 13, 2498. 14597658 10.1101/gr.1239303 PMC403769

[50] E. Gladilin , S. Ohse , M. Boerries , H. Busch , C. Xu , M. Schneider , M. Meister , R. Eils , Sci. Rep. 2019, 9, 7667. 31113982 10.1038/s41598-019-43409-x PMC6529472

[51] Y. Yao , X. Gu , H. Liu , G. Wu , D. Yuan , X. Yang , Y. Song , Br. J. Cancer 2014, 111, 355. 24918821 10.1038/bjc.2014.267 PMC4102939

[52] R. Y. Nguyen , H. Xiao , X. Gong , A. Arroyo , A. T. Cabral , T. T. Fischer , K. M. Flores , X. Zhang , M. E. Robert , B. E. Ehrlich , M. Mak , Commun. Biol. 2022, 5, 202. 35241781 10.1038/s42003-022-03121-5 PMC8894393

[53] S. Werner , L. Keller , K. Pantel , Mol. Aspects Med. 2020, 72, 100817. 31563278 10.1016/j.mam.2019.09.001

[54] Q. Li , R. Wang , Z. Yang , W. Li , J. Yang , Z. Wang , H. Bai , Y. Cui , Y. Tian , Z. Wu , Y. Guo , J. Xu , L. Wen , J. He , F. Tang , J. Wang , Genome Med. 2022, 14, 87. 35962452 10.1186/s13073-022-01089-9 PMC9375433

[55] X. Zhou , W. Wen , X. Shan , W. Zhu , J. Xu , R. Guo , W. Cheng , F. Wang , L.‐W. Qi , Y. Chen , Z. Huang , T. Wang , D. Zhu , P. Liu , Y. Shu , Oncotarget 2017, 8, 6513. 28036284 10.18632/oncotarget.14311 PMC5351649

[56] K. Takezawa , V. Pirazzoli , M. E. Arcila , C. A. Nebhan , X. Song , E. de Stanchina , K. Ohashi , Y. Y. Janjigian , P. J. Spitzler , M. A. Melnick , G. J. Riely , M. G. Kris , V. A. Miller , M. Ladanyi , K. Politi , W. Pao , Cancer Discov. 2012, 2, 922. 22956644 10.1158/2159-8290.CD-12-0108 PMC3473100

[57] J. De Grève , T. Moran , M.‐P. Graas , D. Galdermans , P. Vuylsteke , J.‐L. Canon , D. Schallier , L. Decoster , E. Teugels , D. Massey , V. K. Chand , J. Vansteenkiste , Lung Cancer 2015, 88, 63. 25682316 10.1016/j.lungcan.2015.01.013

[58] C.‐F. Tsai , C.‐C. Hsu , J.‐N. Hung , Y.‐T. Wang , W.‐K. Choong , M.‐Y. Zeng , P.‐Y. Lin , R.‐W. Hong , T.‐Y. Sung , Y.‐J. Chen , Anal. Chem. 2014, 86, 685. 24313913 10.1021/ac4031175

[59] M. C. Chambers , B. Maclean , R. Burke , D. Amodei , D. L. Ruderman , S. Neumann , L. Gatto , B. Fischer , B. Pratt , J. Egertson , K. Hoff , D. Kessner , N. Tasman , N. Shulman , B. Frewen , T. A. Baker , M.‐Y. Brusniak , C. Paulse , D. Creasy , L. Flashner , P. Mallick , Nat. Biotechnol. 2012, 30, 918. 23051804 10.1038/nbt.2377 PMC3471674

[60] S. Tyanova , T. Temu , P. Sinitcyn , A. Carlson , M. Y. Hein , T. Geiger , M. Mann , J. Cox , Nat. Methods 2016, 13, 731. 27348712 10.1038/nmeth.3901

[61] D. Szklarczyk , A. L. Gable , D. Lyon , A. Junge , S. Wyder , J. Huerta‐Cepas , M. Simonovic , N. T. Doncheva , J. H. Morris , P. Bork , L. J. Jensen , C. von Mering , Nucleic Acids Res. 2019, 47, D607. 30476243 10.1093/nar/gky1131 PMC6323986

