# Supplementary material for: Mapping Nanoscale‐To‐Single‐Cell Phosphoproteomic Landscape by Chip‐DIA
Source: Adv Sci (Weinh). 2024 Oct 14;12(1):2402421. doi: 10.1002/advs.202402421 (PMC11714195; doi:10.1002/advs.202402421)
Supplement: Supplementary file 1 — Supporting Information [file ADVS-12-2402421-s008.docx]

**Supporting Information**

(Figures S1–S13 and Tables S1–S2)

**Mapping Nanoscale-To-Single-Cell Phosphoproteomic Landscape by Chip-DIA**

*Gul Muneer^[a,b,c]+^, Sofani Tafesse Gebreyesus^[a]+^, Ciao-Syuan Chen^[a]^, Tzu-Tsung Lee^[a]^, Fengchao Yu^[d]^, Chih-An Lin^[e]^, Min-Shu Hsieh^[f]^, Alexey I. Nesvizhskii^[d,g]^, Chao-Chi Ho^[e]^, Sung-Liang Yu^[h]^, Hsiung-Lin Tu^[a,c,i,j]^*, Yu-Ju Chen^[a,c,i,k]^**

[a] G. Muneer, S. T. Gebreyesus, C-S. Chen, T-T. Lee, H-L. Tu, Y-J. Chen
Institute of Chemistry, Academia Sinica, Taipei, 115201, Taiwan
E-mail: hltu@gate.sinica.edu.tw; yujuchen@gate.sinica.edu.tw

[b] G. Muneer
Institute of Biochemical Sciences, National Taiwan University, Taipei, 106319, Taiwan

[c] G. Muneer, H-L. Tu, Y-J. Chen

Chemical Biology and Molecular Biophysics Program, Taiwan International Graduate Program, Academia Sinica, Taipei, 11529, Taiwan

[d] F. Yu, A. I. Nesvizhskii
Department of Pathology, University of Michigan, Ann Arbor, Michigan, 48109, USA

[e] C-A. Lin, C-C. Ho

Department of Internal Medicine, National Taiwan University Hospital, Taipei, 10051, Taiwan

[f] M-S. Hsieh

Department of Pathology, National Taiwan University Cancer Center, Taipei, 10617, Taiwan

Department of Pathology, National Taiwan University Hospital, Taipei, 100225, Taiwan

Graduate Institute of Pathology, National Taiwan University College of Medicine, Taipei, 10051, Taiwan

[g] A. I. Nesvizhskii

Department of Computational Medicine and Bioinformatics, University of Michigan, Ann Arbor, Michigan, 48109-2218, USA

[h] S-L Yu

Department of Clinical Laboratory Science and Medical Biotechnology, College of Medicine, National Taiwan University, Taipei, 10048, Taiwan

Department of Laboratory Medicine, National Taiwan University Hospital, Taipei, 10002, Taiwan

[i] H-L. Tu, Y-J. Chen

Genome and Systems Biology Degree Program, Academia Sinica and National Taiwan University, Taipei, 10617, Taiwan

[j] H-L. Tu

Nano Science and Technology Program, Taiwan International Graduate Program, Academia Sinica, Taipei, 11529, Taiwan

[k] Y-J. Chen

Department of Chemistry, National Taiwan University, Taipei, 10617, Taiwan

+These authors contributed equally to this work.

*Corresponding authors.

**Table of Contents**

[Figures S1–S13 3](#_Toc173537028)

[Tables S1–S2 17](#_Toc173537030)

# Figures S1–S13

#
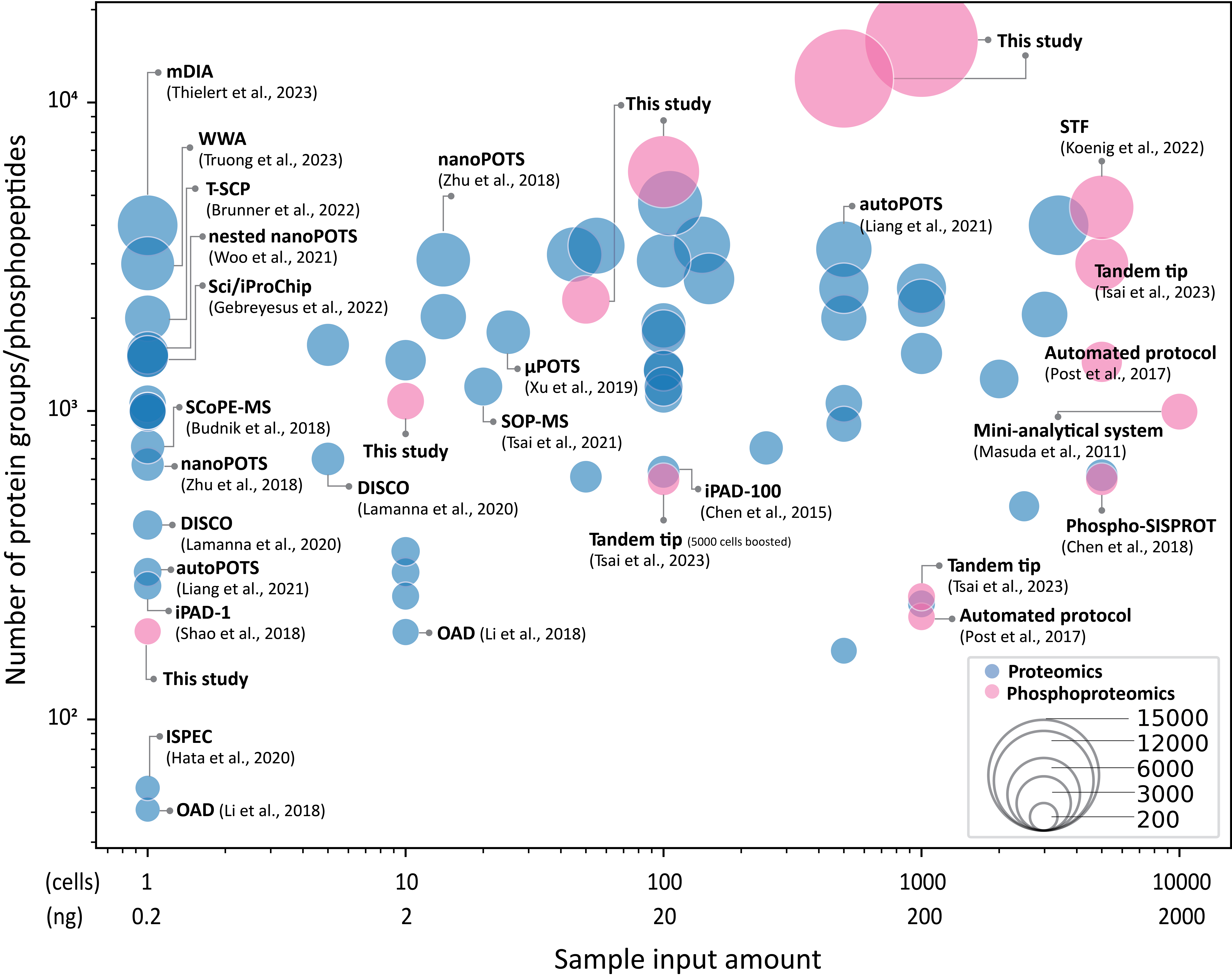


**Figure S1. Comparative summary illustrating sample input and identification coverage across recent proteomic and phosphoproteomic approaches.** The figure shows the performance sensitivity, represented by the number of proteins/phosphopeptides (indicated by circles of different sizes) identified per nanogram (ng) of sample across different input amounts.


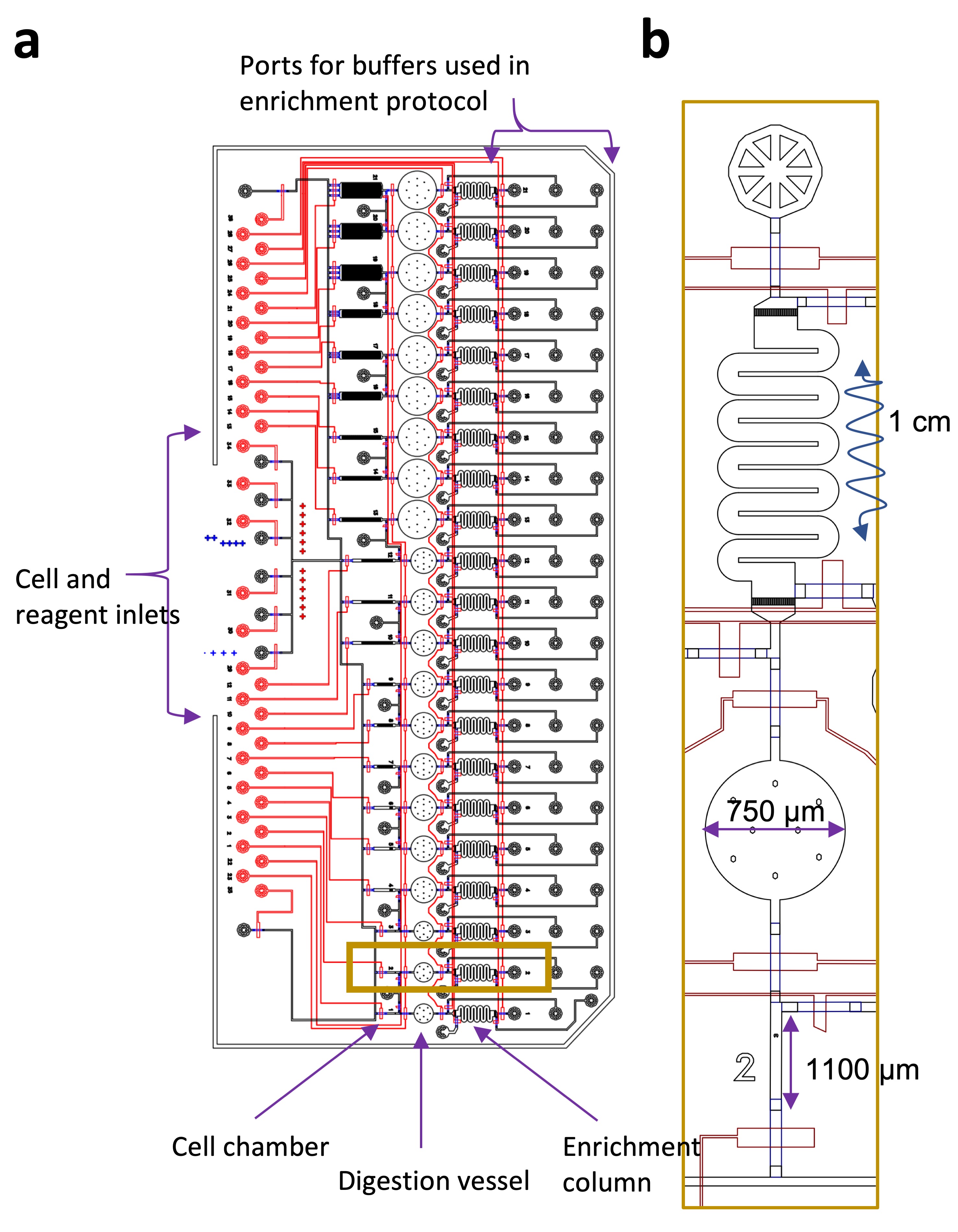


**Figure S2. Chip design and dimensions**. (a) The iPhosChip layout. The flow layer (black and blue) consists of 18 units for processing 1, 10, 50, 100, 500, and 1000 cells (in triplicate) from bottom to top. Each unit is composed of cell and reagent inlets, a cell chamber, a digestion vessel, and an enrichment column. The control layer, shown in red, contains 36 actuation valves. (b) A zoom-in view of a single operational unit for handling the single-cell phosphoproteomic workflow.

**
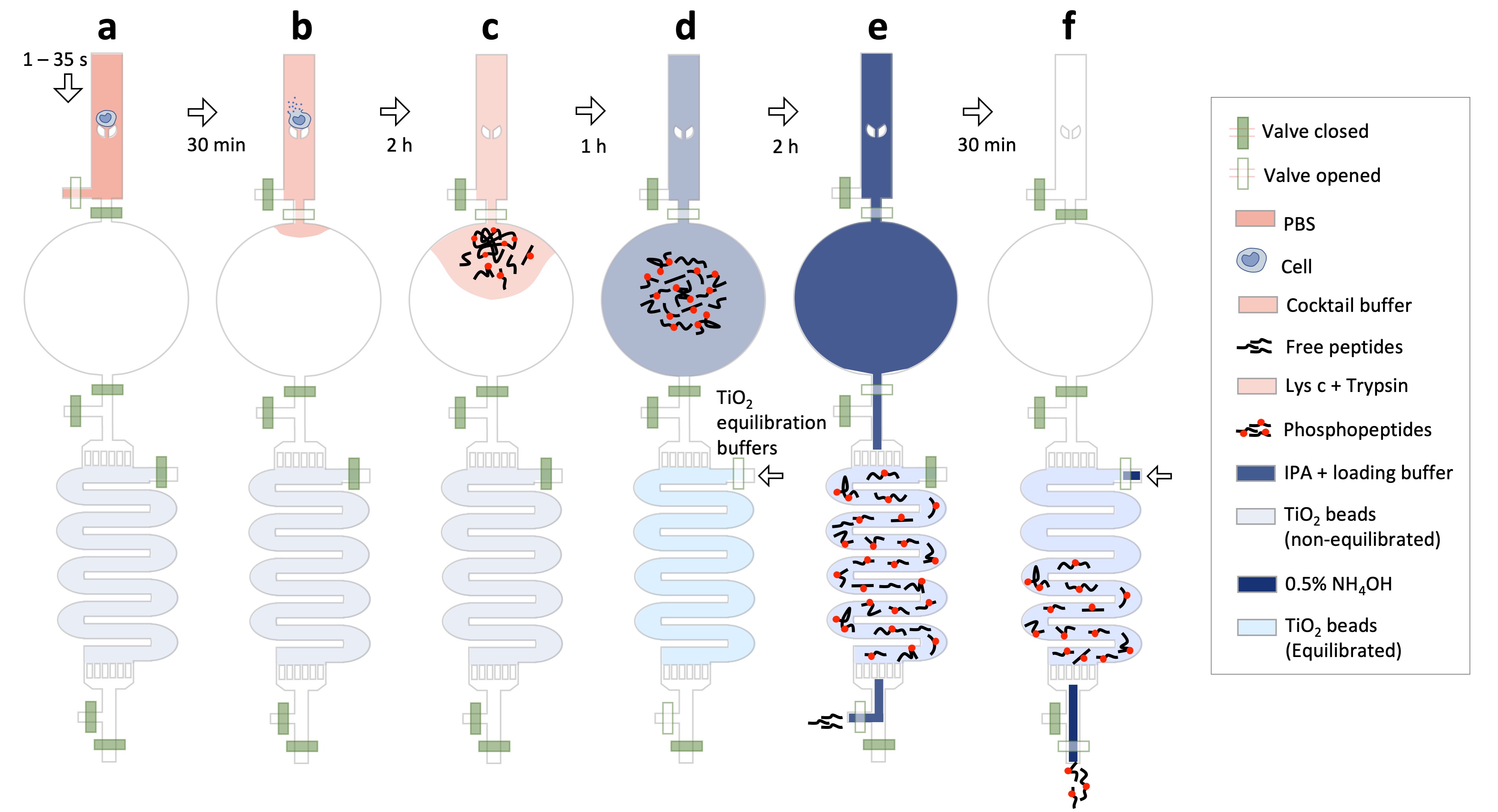
**

**Figure S3. Operational procedure of the sample preparation workflow using iPhosChip**. (a) Cell trapping and imaging. (b) One-step cell lysis and simultaneous protein reduction and alkylation using a cocktail lysis buffer. (c) Enzymatic protein digestion. (d) Loading of buffer C (3.2 M lactic acid, 60% ACN, 0.1% TFA) for equilibration of TiO_2_ beads in the enrichment columns. (e) Loading of the peptide mixture, followed by infusion of IPA and enrichment buffer (3.2 M lactic acid, 60% ACN, 1% TFA) for phosphopeptide enrichment in the TiO_2_ columns. (f) Elution of the phosphopeptides. Overall, the entire sample preparation workflow takes approximately 7 h.

**
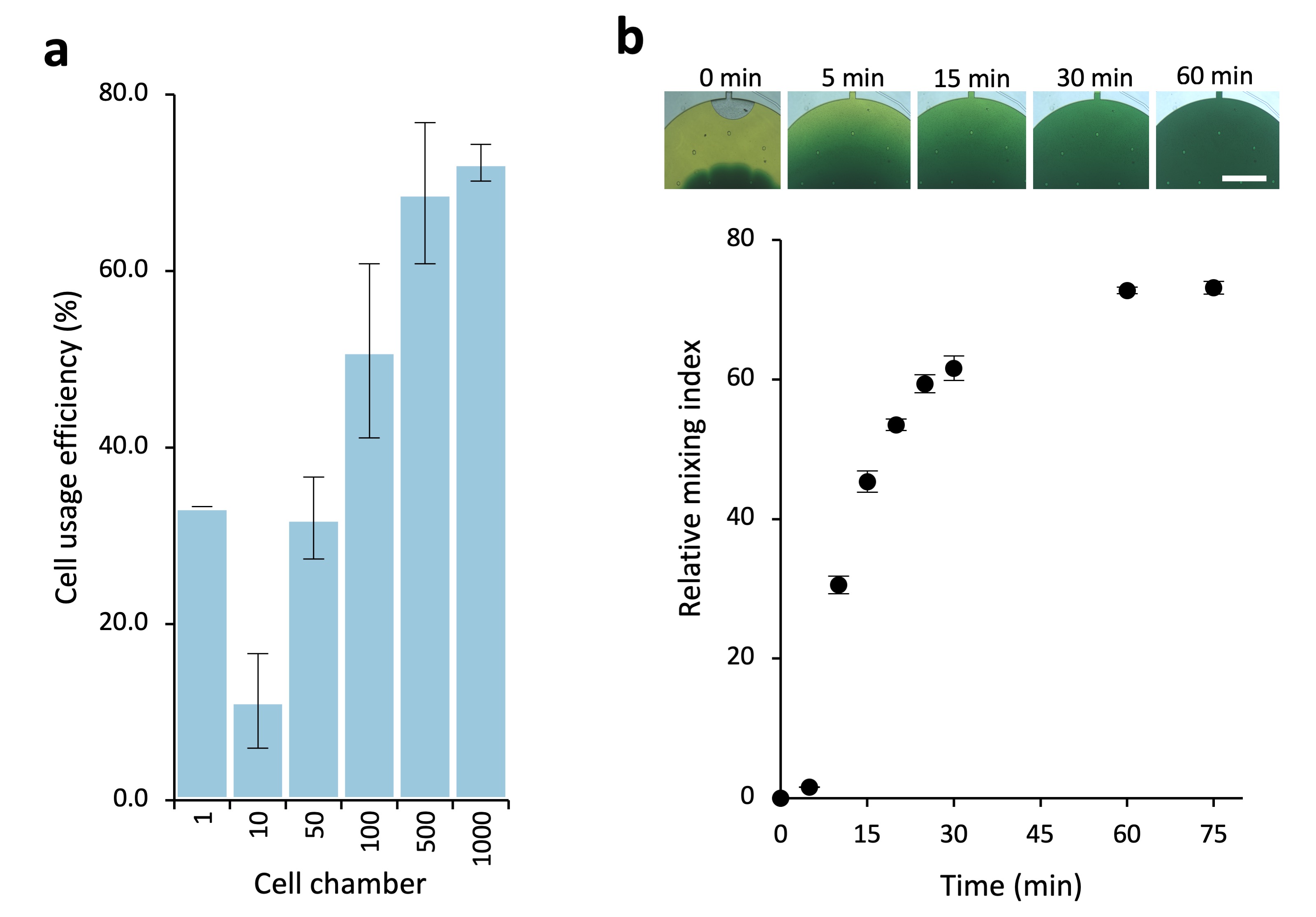
**

**Figure S4. Performance characterization of functional modules within iPhosChip.** (a) Cell usage efficiency in various cell chambers which was determined by using 5, 10, and 30 μL of cell solutions containing 150, 300 and 900-1800 cells for the 1-10, 50-100, and 500-1000 cell chambers, respectively. Data are presented as mean values ± SD obtained from 3 independent experiments. (b) Evaluation of mixing efficiency within the digestion vessel (530 nL) of iPhosChip by shaking (via a plate shaker). Data are presented as mean values ± SD obtained from 3 independent experiments. The upper panel shows the representative time-lapse images of a reaction vessel filled with buffers used for the workflow containing blue and yellow dyes. Scale bar: 750 μm. Source data are provided as a Source Data file.

**
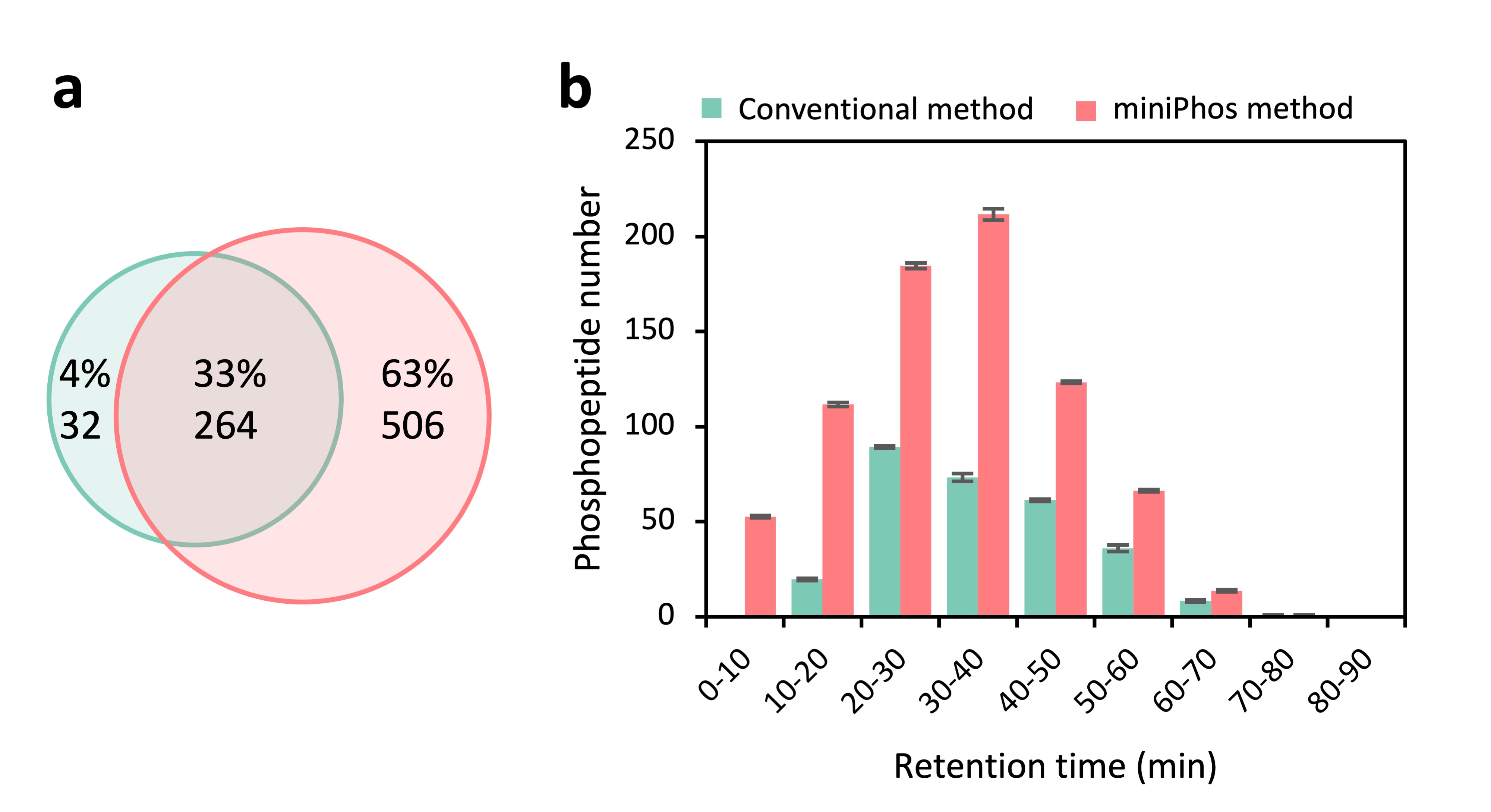
**

**Figure S5. Comparison of phosphoproteomic profiling of 50 PC9 cells using conventional and miniaturized workflows (miniPhos) in iPhosChip.** (a) Overlap of identified phosphopeptides between the conventional workflow in iPhosChip (296 phosphopeptides) and the miniaturized workflow in iPhosChip (770 phosphopeptides). (b) Distribution of retention time of detected phosphopeptides from both methods. All the data are shown as mean ± SD from 3 independent experiments. Source data are provided as a Source Data file.


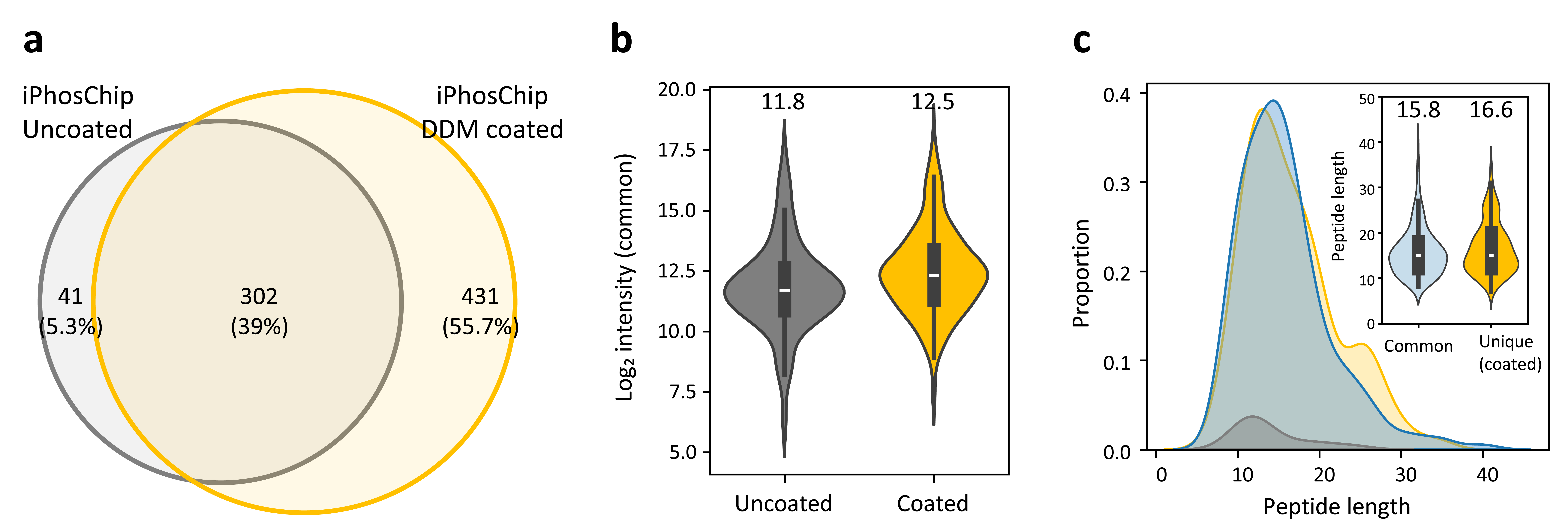


**Figure S6. Effect of DDM-coating on phosphopeptide recovery using iPhosChip.** (a) Overlap of phosphopeptides detected from 50 PC9 cells processed in DDM-coated iPhosChip versus uncoated iPhosChip. (b) Violin plot showing the intensity distribution of commonly quantified phosphopeptides by each method. (c) Distribution of peptide length for phosphopeptides that are commonly identified and those uniquely identified by both workflows. Source data are provided as a Source Data file.


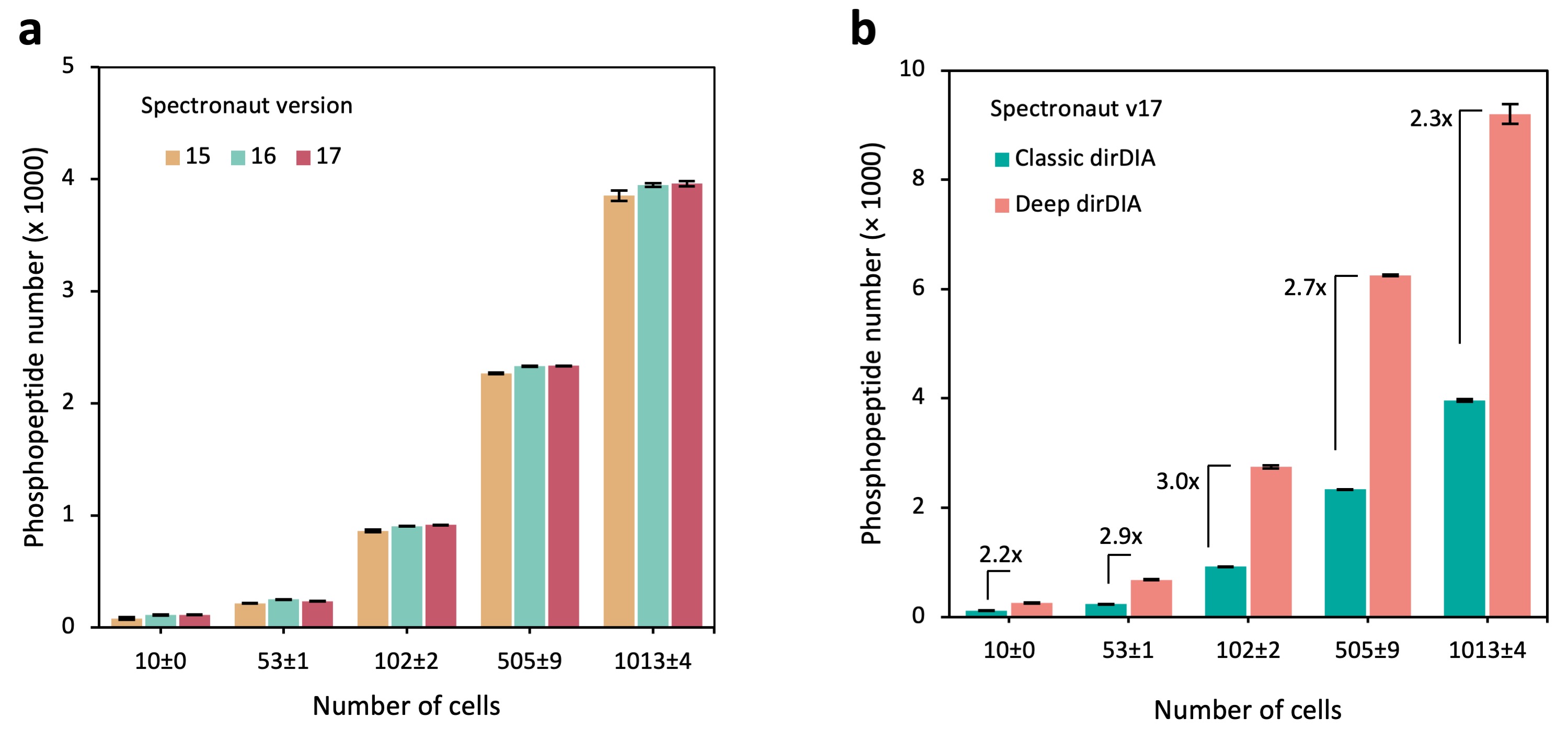


**Figure S7. Comparison of identification performance across different versions and modes in Spectronaut.** (a) Summary of identified phosphopeptides from 10–1000 cells using the classic directDIA mode across different versions of Spectronaut (n = 3 independent cell samples for each condition). (b) Summary of identified phosphopeptides from 10–1000 cells using the classic directDIA and deep directDIA+ modes in Spectronaut v17 (*n* = 3 independent cell samples for each condition). All the data are shown as mean ± SD from 3 independent experiments. Source data are provided as a Source Data file.


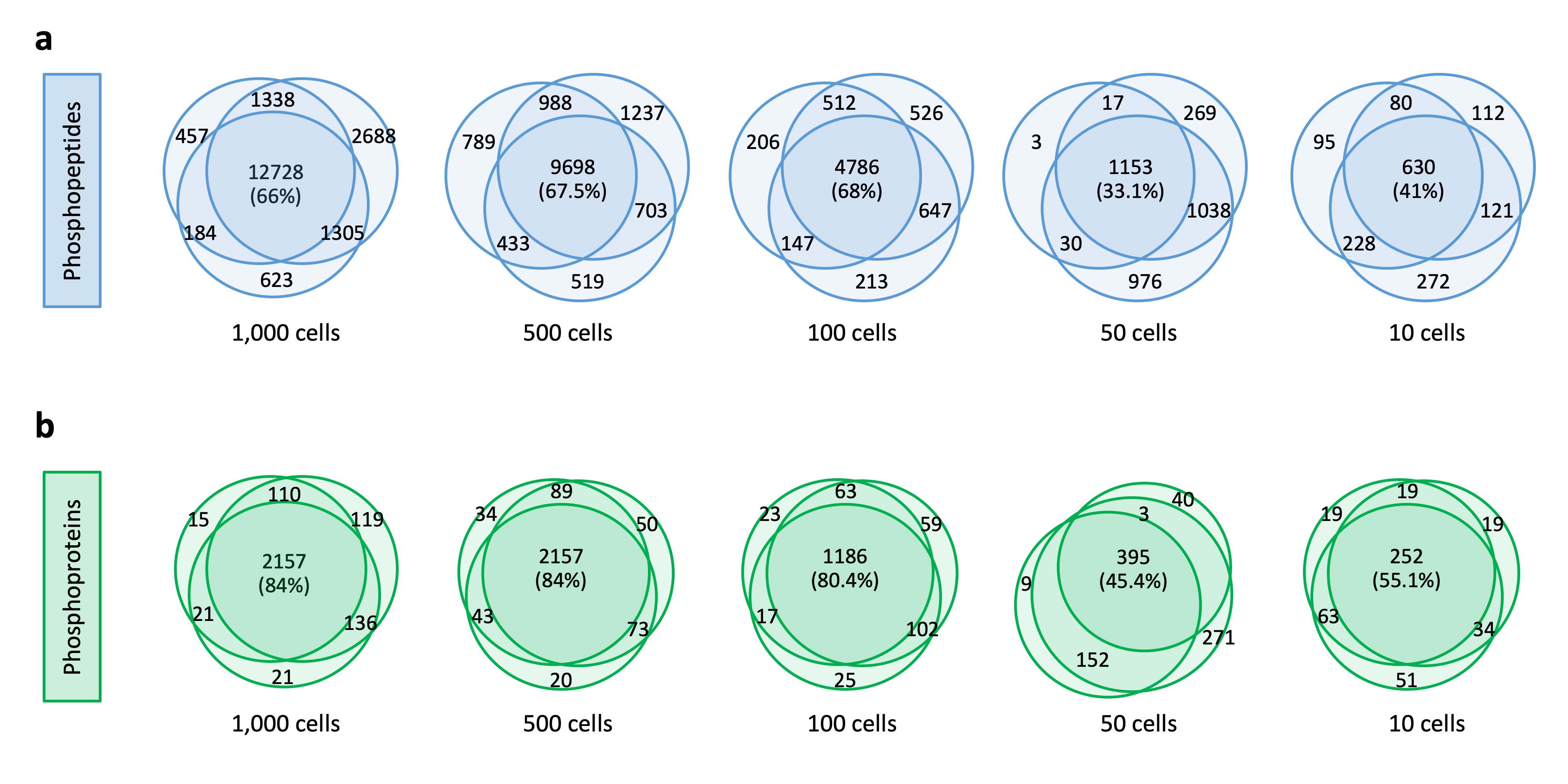


**Figure S8. Commonly identified phosphopeptides and phosphoproteins in triplicate analyses from different PC9 cell numbers.** Overlaps of identified (a) phosphopeptides and (b) phosphoproteins across triplicate analyses of varying cell numbers.


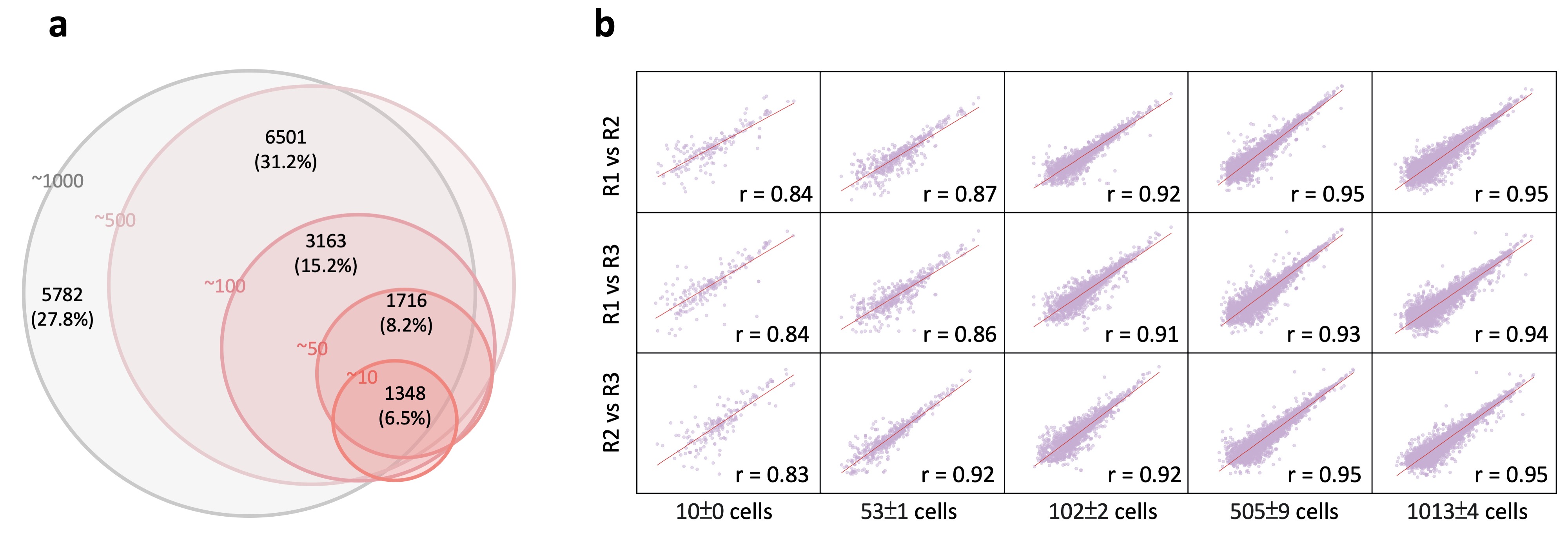


**Figure S9. Assessment of commonly identified phosphopeptides and quantification reproducibility in triplicate analyses of different PC9 cell numbers.** (a) Overlap of identified phosphopeptides across 10–1000 cells. (b) Pearson’s correlation coefficients of quantified phosphopeptides across different cell loadings. Source data are provided as a Source Data file.


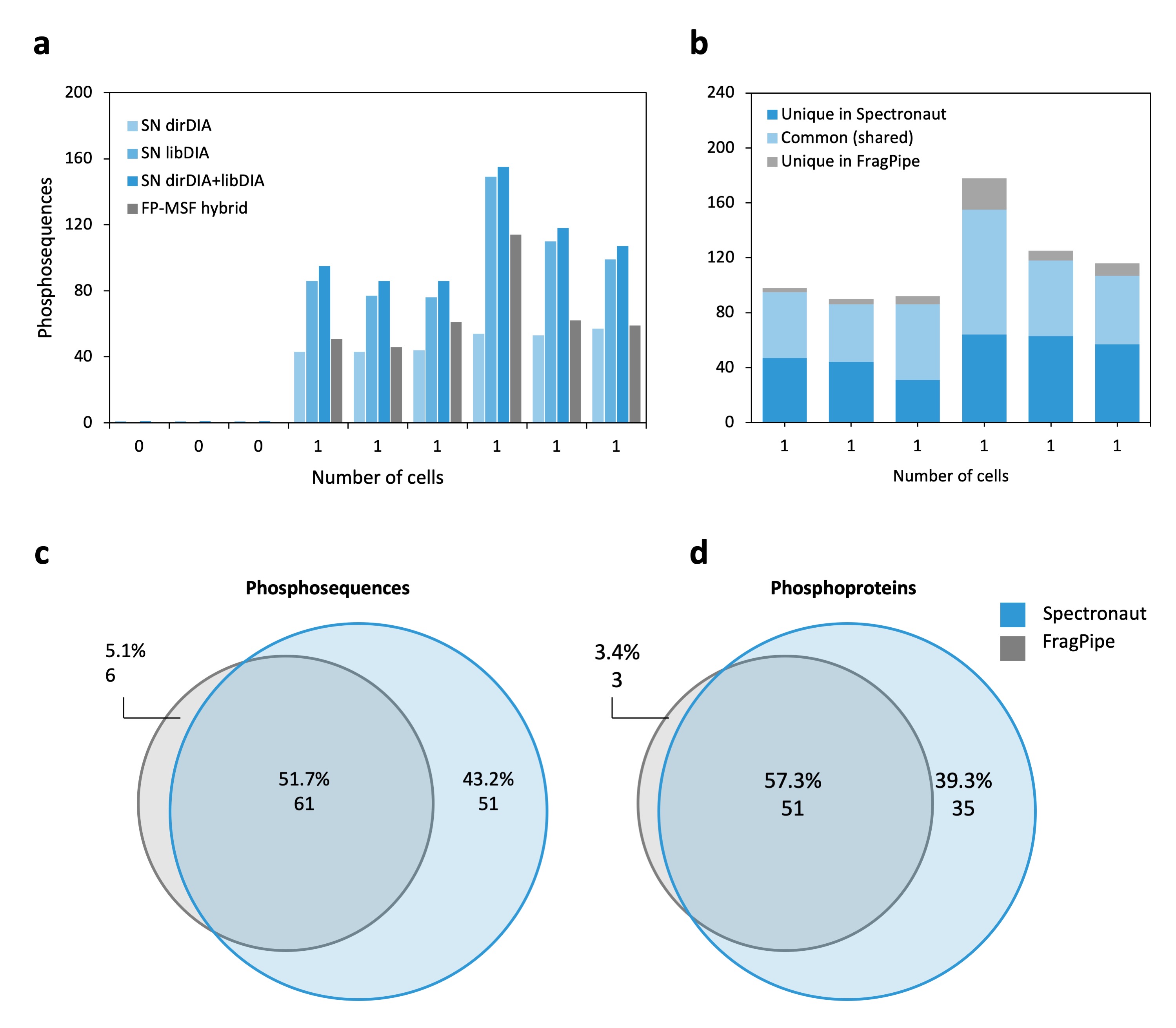


**Figure S10. Comparison of phospho-sequences identified in six single-cell replicates by FragPipe and Spectronaut.** (a) Number of unique phospho-sequences identified by Spectronaut’s dirDIA, libDIA, integrated dirDIA and libDIA, and FragPipe’s hybrid DIA. (b) Total number of phospho-sequences identified by Spectronaut and FragPipe. (c) Overlap of phospho-sequences identified by FragPipe and Spectronaut. (d) Overlap of phosphoproteins identified by FragPipe and Spectronaut. SN: Spectronaut; FP: FragPipe. Source data are provided as a Source Data file.


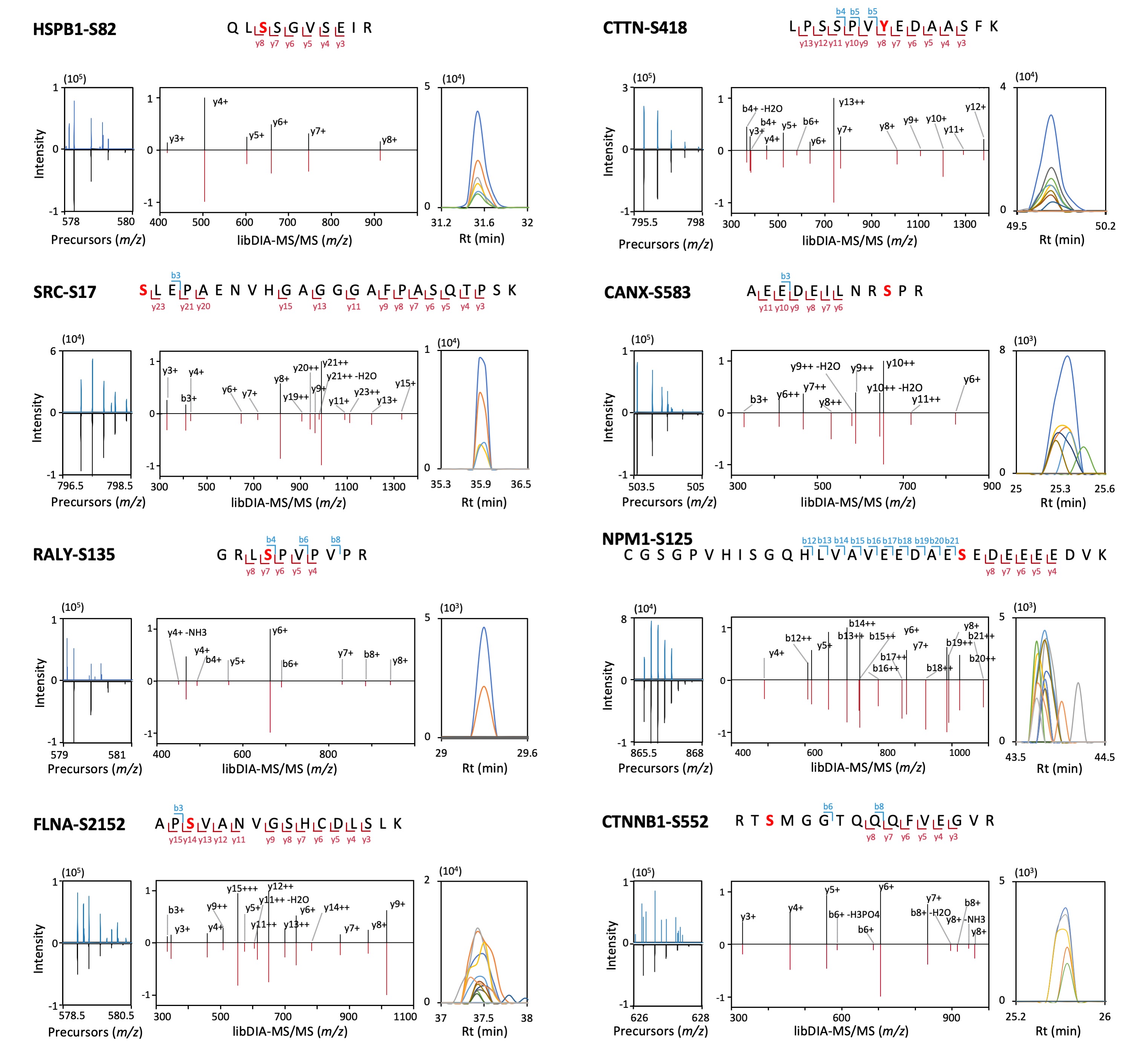


**Figure S11.** **Summary of precursors monoisotopic distribution, fragment ion spectra and extracted ion chromatogram from exemplary cancer-related phosphopeptides identified in single-cells using libDIA.** Representative spectra identified from libDIA include: raw monoisotopic pattern distribution of precursor (left), fragment spectral similarity between the sample and library (middle), and extracted ion chromatogram of indicated phosphopeptides in single-cell samples (right).





**Figure S12. Summary of quantified phosphopeptides and phosphoproteins in triplicate analyses from 10 cells, 100 cells and 500 cells across three patient-derived lung cancer cell lines.** (a) Number of phosphopeptides quantified in the CLH157, CLH206 and CLH217 from ~10, ~100, and ~500 cells. (b) Overlaps of the quantified phosphopeptides and phosphoproteins in triplicate analyses across different cell loadings. All the data are shown as mean ± SD from 3 independent experiments. Source data are provided as a Source Data file.


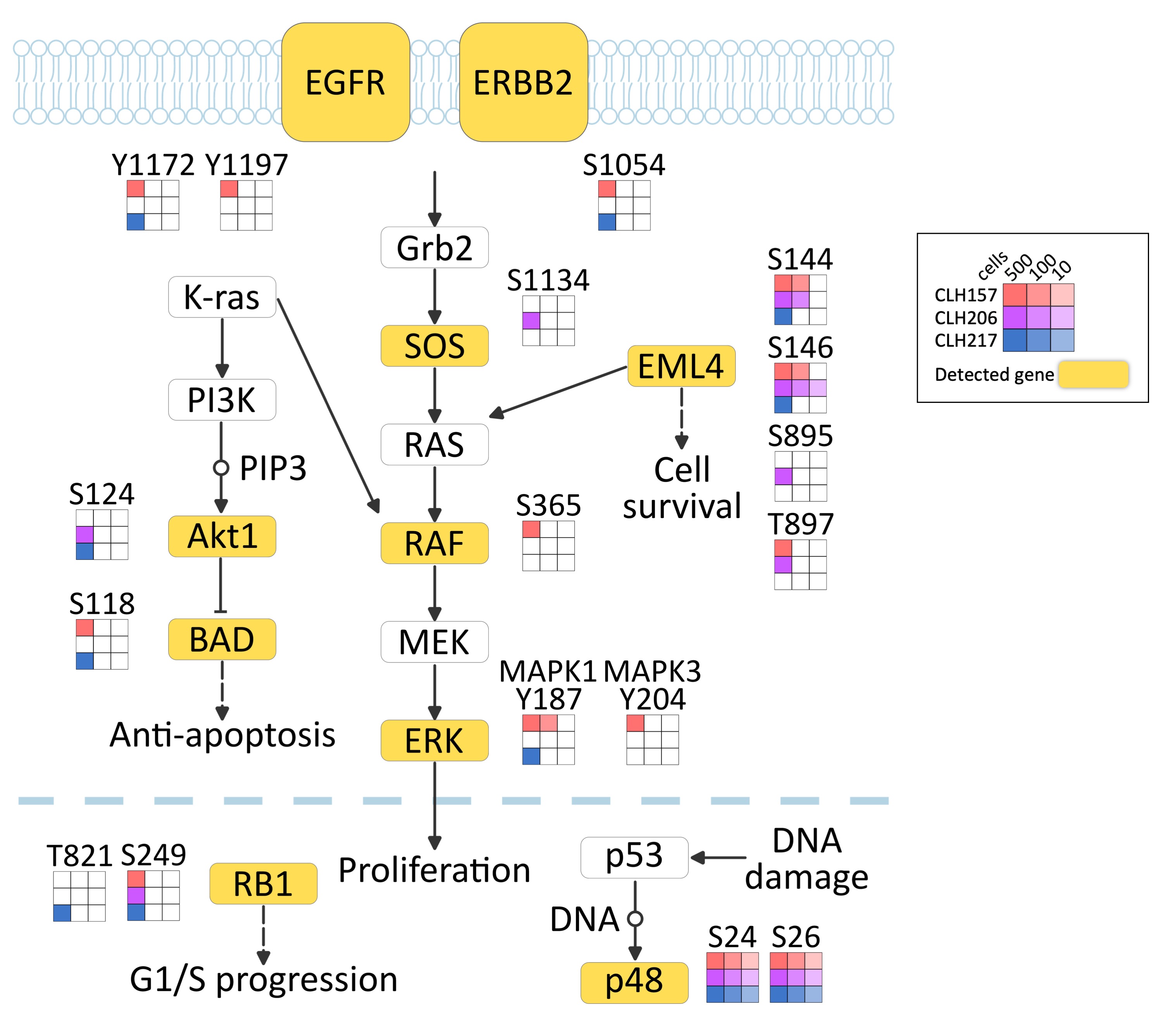


**Figure S13. Phosphosites in the non-small cell lung cancer (NSCLC) pathway across different patient-derived cell lines.** Mapping coverage of phosphosites and phosphoprotein in the NSCLC pathway from three patient-derived lung cancer cell lines (CLH157, CLH206 and CLH217) at different cell numbers.

# Tables S1–S2

**Table S1. Summary of phosphoproteome coverages in spectral libraries.** This table includes the library input amount, number of total peptide precursors, phosphopeptides, and phosphoproteins in each generated library.

| **Library No.** | **Library input** | **Precursors** | **Phosphopeptides** | **Phosphoproteins** |
| --- | --- | --- | --- | --- |
| **Lib 1** | 10 ng (50 cells) | 775 | 688 | 266 |
| **Lib 2** | 20 ng (100 cells) | 3,027 | 2,775 | 740 |
| **Lib 3** | 100 ng (500 cells) | 6893 | 6291 | 1364 |
| **Lib 4** | 200 ng (1000 cells) | 8725 | 7956 | 1657 |
| **Lib 5** | 500 ng (2500 cells) | 17060 | 15587 | 2439 |
| **Lib 6** | 1000 ng (5000 cells) | 33316 | 29621 | 3659 |
| **Lib 7** | 1500 ng (7500 cells) | 34174 | 30374 | 3729 |

**Table S2. Phosphotyrosine proteins identified in single-cell samples.** This table summarizes the gene name, description of proteins, and tyrosine site information identified at the single-cell level.

| **Genes** | **Protein descriptions** | **Protein groups** | **PTM site** |
| --- | --- | --- | --- |
| USE1 | Vesicle transport protein USE1 | Q9NZ43 | Y61 |
| PPP1R12A | Protein phosphatase 1 regulatory subunit 12A | O14974 | Y446 |
| SRSF9 | Serine/arginine-rich splicing factor 9 | Q13242 | Y214 |
| KRT18 | Keratin, type I cytoskeletal 18 | P05783 | Y36 |
| BCLAF1 | Bcl-2-associated transcription factor 1 | Q9NYF8 | Y284 |
| MCM2 | DNA replication licensing factor MCM2 | P49736 | Y137 |
| PLEC | Plectin | Q15149 | Y4393 |
| CTTN | Src substrate cortactin | Q14247 | Y421 |
